# Multi-output prediction of dose–response curves enables drug repositioning and biomarker discovery

**DOI:** 10.1038/s41698-024-00691-x

**Published:** 2024-09-20

**Authors:** Juan-José Giraldo Gutierrez, Evelyn Lau, Subhashini Dharmapalan, Melody Parker, Yurui Chen, Mauricio A. Álvarez, Dennis Wang

**Affiliations:** 1 National Heart and Lung Institute, Imperial College London, London, UK; 2 Department of Computer Science, The University of Sheffield, Sheffield, UK; 3 Institute for Human Development and Potential, Agency for Science Technology and Research (A*STAR), Singapore, Republic of Singapore; 4 Nuffield Department of Clinical Medicine, University of Oxford, John Radcliffe Hospital, Oxford, UK; 5 Big Data Institute at the Li Ka Shing Centre for Health Information and Discovery, University of Oxford, Oxford, UK; 6 Department of Mathematics, National University of Singapore, Singapore, Republic of Singapore; 7 Department of Computer Science, The University of Manchester, Manchester, UK; 8 Bioinformatics Institute (BII), Agency for Science Technology and Research (A*STAR), Singapore, Republic of Singapore

**Keywords:** High-throughput screening, Drug development

## Abstract

Drug response prediction is hampered by uncertainty in the measures of response and selection of doses. In this study, we propose a probabilistic multi-output model to simultaneously predict all dose–responses and uncover their biomarkers. By describing the relationship between genomic features and chemical properties to every response at every dose, our multi-output Gaussian Process (MOGP) models enable assessment of drug efficacy using any dose–response metric. This approach was tested across two drug screening studies and ten cancer types. Kullback-leibler divergence measured the importance of each feature and identified *EZH2* gene as a novel biomarker of BRAF inhibitor response. We demonstrate the effectiveness of our MOGP models in accurately predicting dose–responses in different cancer types and when there is a limited number of drug screening experiments for training. Our findings highlight the potential of MOGP models in enhancing drug development pipelines by reducing data requirements and improving precision in dose–response predictions.

## Introduction

Disease heterogeneity poses an enduring challenge in developing effective treatments. Each ailment, be it cancer or other diseases, exhibits distinct molecular and physiological characteristics, demanding tailored therapeutic strategies^1–5^. Meticulously assessing chemical compounds in different cancers with specific disease targets, drug screening provides a comprehensive framework to identify promising candidates for further investigation. When the data is analysed alongside the treated sample’s molecular characteristics and the drug’s chemical properties, the mechanisms of action and biomarkers of response are uncovered, facilitating the design of optimised drug candidates. By implementing large-scale screening platforms and computational models, researchers can systematically evaluate approved drugs, expediting the identification of novel therapeutic applications^2,6,7^.

More recently, machine learning (ML) models played a pivotal role in making drug screening more efficient by predicting drug response^8–11^. For instance, regression models, including support vector machine^12^ and elastic net^13,14^ are applied to predict the drug response by identifying novel molecular markers. Matrix factorisation methods analyse similarities between drugs and cancer cell lines to predict drug response^15–17^. Deep learning methods, owing to their ability to handle high-dimensional features and capture non-linear relationships, have shown great promise in predicting drug responses^9^. Convolutional Neural Networks and Autoencoders have been employed to derive cell line embeddings from multi-omics data^15,16^. Simultaneously, Graph Neural Networks (GNNs) have been utilised to represent drug chemical features by conceptualising drugs as chemical graphs^17–19^. However, these methods require the user to choose a summary metric of dose–response, such as IC50 and AUC, to evaluate and they do not predict the responses at all doses tested^20^. Functional Random Forest^21^ predicts dose–response curves but faces computational constraints and may not capture significant variations due to its smoothing approach. Another limitation of the current ML models is that their performance often diminishes in cross-study tests, where training and testing are done on different datasets, due to overfitting and inconsistency in drug response profiling^22^. These limitations are difficult to overcome because of inherent noise and variability in the drug screening experiments. Supplementary Table 1 compares the attributes of our model to other ML drug-prediction methods.

Model interpretation and identification of the most predictive features of drug sensitivity are important for translation to patients. ML models have identified biomarkers of response, such as the EGFR mutations linked to lapatinib sensitivity^23^, by measuring feature importance with Shapley values^24,25^. Harun et al.^25^ applied the bootstrap analysis to determine the confidence intervals around SHAP values derived from their XGBoost model, which was developed to quantify the exposure-response relationship. These approaches are also limited by uncertainty in the dose–response measures and molecular profiling. Therefore, it is crucial to provide confidence intervals and estimate the probability of biomarkers. We previously introduced a probabilistic framework with a Gaussian Process (GP) model to address variability from experimental standards and curve fitting uncertainties^11^. Though GPs are typically used for non-linear regression with a focus on single-output, they have benefits in multi-output regression. Instead of treating outputs independently, integrating their correlations directly into the GP model enhances predictive accuracy, especially with limited samples. Multi-output GP (MOGP) models have been successfully used for prediction in spatio-temporal datasets^26^, sensor networks^27^, robotics^27,28^, among other fields. They have also been used in dose–response modelling, but only for extrapolating curves and not for describing the relationship with molecular biomarkers or drug chemistry^29^.

In this study, we show that MOGP can be used to model dose–responses using genomic features of samples and the chemical features of the drugs used in vitro experiments. Unlike previous studies that modelled summary statistics extracted from dose–response curves, we built MOGP models to describe the responses at all doses, which enables the assessment of any dose–response summary statistic. We also introduce a novel approach for detecting biomarkers of response in MOGPs by comparing probability distributions and show its robustness in two pharmacogenomic drug screens. Finally, we show that MOGP can accurately predict dose–responses in multiple cancer types with minimal training data.

## Results

### Multi-Output Gaussian Process (MOGP) models describe drug response at multiple doses

**Fig. 1:**
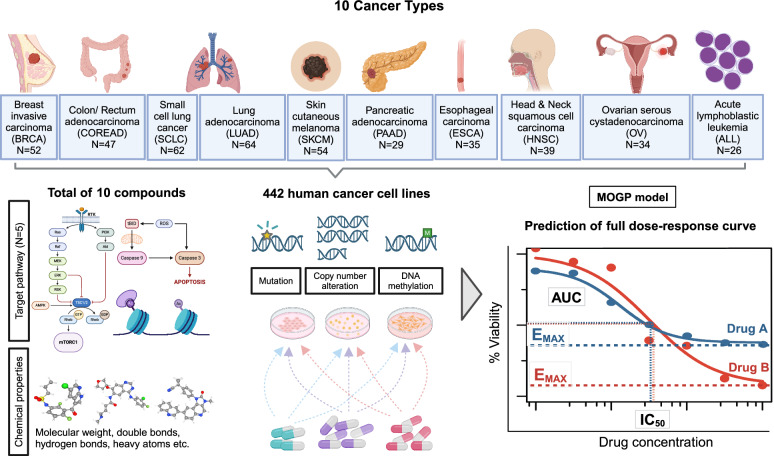
Overview of experiment to train and test machine learning prediction of dose-response across 10 cancer types. Two datasets, GDSC1 (only SKCM cell lines; *N* = 52) and GDSC2, were constructed by consolidating dose–response data for ten drugs (Vorinostat, Axitinib, *PLX-4720, MK-2206, Pictilisib, AZD8055, *SB590885, TW 37, Trametinib and *Dabrafenib) across 442 human cancer cell lines, sourced from the GDSC database. These ten drugs target pathways including ERK/MAPK signalling, PI3K/MTOR signalling, RTK signalling, apoptosis regulation and chromatin histone acetylation. Dose–response data for drugs marked with an asterisk (*) were obtained from both GDSC1 and GDSC2, while data for the remaining drugs are from GDSC2 only. Both datasets consist of multiple cancer cell lines (*N* = number of unique cell lines) representing each of ten different cancer types (BRCA, COREAD, SCLC, LUAD, SKCM, PAAD, ESCA, HNSC, OV and ALL). Molecular features characterising these cell lines (genetic variations, copy number alterations, DNA methylation) and the chemical properties of these drugs (sourced from PubChem) were also included. These comprehensive datasets served as input for the MOGP model for estimating the relative importance of these input features based on KL divergence and predicting full dose–response curves. Prediction of a full dose–response curve enables the extraction of various drug response metrics such as Emax, IC50 and area under the dose–response curve (AUC), which describe and quantify the potency and efficacy of different drugs. Differences in their maximum achievable effect (Emax) can be observed despite having similar potency and overall activity (similar IC50 and AUC values) (Supplementary Fig. 1). Understanding this distinction is crucial for determining the most appropriate drug that offers the most significant therapeutic benefits while minimising adverse effects and achieving the desired therapeutic outcomes. Figure was created with BioRender.

We retrieved dose–response and genomics data from the Genomics of Drug Sensitivity in Cancer (GDSC), which included dose–responses obtained from the treatment of ten drugs: Vorinostat, Axitinib, PLX-4720, MK-2206, Pictilisib, AZD8055, SB590885, TW 37, Trametinib, and Dabrafenib, on 442 human cancer cell lines representing ten distinct cancer types (Fig. 1). These cancer types were among those with the largest response sample size when tested with these ten drugs in the GDSC datasets.

**Fig. 2:**
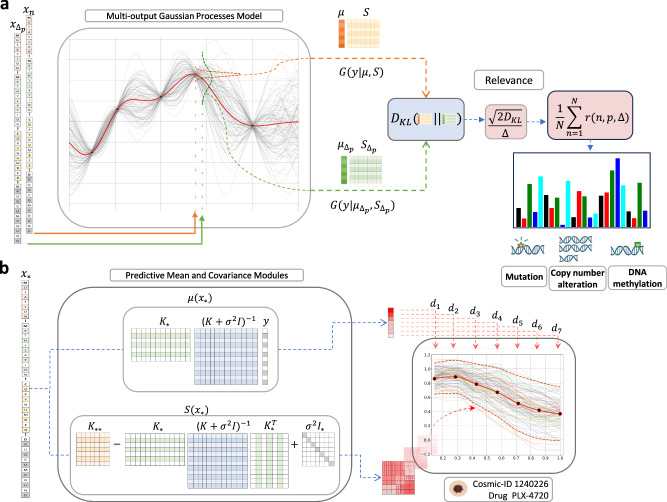
Overview of the multi-output model for identifying biomarkers and predicting dose-response. **a** Kullback-Leibler relevance determination to estimate feature importance. To compute the relevance of a feature w.r.t a data observation we have to make two predictions, one for the original observation $x$, and another where such an observation is subtly modified by a small $\varDelta$ on the $p$-th feature, ${x}_{{\varDelta }_{p}}$. The MOGP outputs two distributions, one for input $x$ and another for ${x}_{{\varDelta }_{p}},$ then the ${D}_{{KL}}[.{||}.]$ module computes a Kullback-Leibler divergence between both predictive distributions and then a normalisation applies the operation $\sqrt{2{D}_{{KL}}[.{||}.]}/\varDelta$ to obtain the relevance of the $p$-th feature (see section Kullback-Leibler Relevance Determination for additional details). **b** Prediction of full dose–response curves using MOGP. The input vector ${x}_{* }$ is composed of: the cell line genomic features, mutation, methylation and copy number, and the drug compounds. The vector ${x}_{* }$ feeds two blocks of the MOGP prediction, such blocks generate a predictive Gaussian distribution, ${G(y}_{* }|\mu ({X}_{* }),S({X}_{* }))$, with mean $\mu ({x}_{* })$ (red-ish vector) and covariance $S({x}_{* })$ (red-ish matrix); both blocks are a pictorial representation of Eqs. (3) and (4). The last panel to the right hand side shows the prediction of a melanoma cancer cell line (COSMIC ID: 1240226) from the GDSC2 dataset and treated with PLX-4720. The mean vector $\mu ({x}_{* })$ has a size of $D=7$, each of its entries represent the cell viability of the $d$-th drug concentration (black dots). The covariance matrix $S({x}_{* })$ encodes the uncertainty of the prediction; it can (loosely) be expressed as the dashed red line that accounts for a confidence interval of two standard deviations. The multiple coloured functions amongst the dashed red line depict random samples taken from the predictive Gaussian distribution to exemplify the stochastic nature of the MOGP prediction.

Given the promising clinical outcomes observed with BRAF inhibitor (BRAFi) therapy in BRAF-mutated melanoma, we directed our initial focus to the dose–response profiles associated with inhibitor compounds targeting the BRAF kinase within the ERK/MAPK pathway. Specifically, we selected dose–response data from the treatment of three BRAF-targeted drugs: i) Dabrafenib, ii) PLX-4720 and iii) SB590885 across multiple cancer cell lines from GDSC1 (comprising only skin cutaneous melanoma (SKCM) cell lines; *N* = 52) and GDSC2 (*N* = 279) datasets. All SKCM cell lines from the GDSC1 were also incorporated in the drug screening process of GDSC2, thus also among the cell lines included in the GDSC2 dataset used in this study. Cell lines represent the top five cancer types with the largest response sample size when tested with these three drugs in the GDSC2 datasets: breast invasive carcinoma (BRCA), colon/rectum adenocarcinoma (COREAD), small cell lung cancer (SCLC), lung adenocarcinoma (LUAD) and SKCM. Three types of molecular features; genetic variations in high-confidence cancer genes, copy number alteration (CNA) status of recurrent altered chromosomal segments (RACSs), and DNA methylation status of informative CpG islands (iCpGs) for these cancer cell lines were also extracted from the GDSC database, along with chemical features specific to the aforementioned three drugs from the PubChem databases. Using a multi-output Gaussian process (MOGP) model together with a ranking features method based on the Kullback-leibler (KL) divergence (Fig. 2a), we elucidate the relationships between genomic features and drug response curves of SKCM cell lines treated with these three BRAF-targeted drugs from both GDSC1 (*N* = 52) and GDSC2 (*N* = 54) datasets. The MOGP models the cell viabilities for various dose concentrations as outputs, while the KL method uses the MOGP predictions to determine the relevance (or importance) of the features. Our proposed KL relevance method was compared against the ANOVA analysis performed in the GDSC database, focusing on 24 melanoma-specific features (ANOVA analysis considered only mutation and CNA features). Additionally, we used the trained MOGP models to predict dose-specific response in new experiments (Fig. 2b). We assessed the prediction performance across cell lines from BRCA, COREAD, LUAD, melanoma, and SCLC cancers in GDSC2, examining model performance when trained across different cancer types and with increasing training sample sizes.

### Biomarkers of BRAF inhibitor response identified by their relevance in MOGP models

**Fig. 3:**
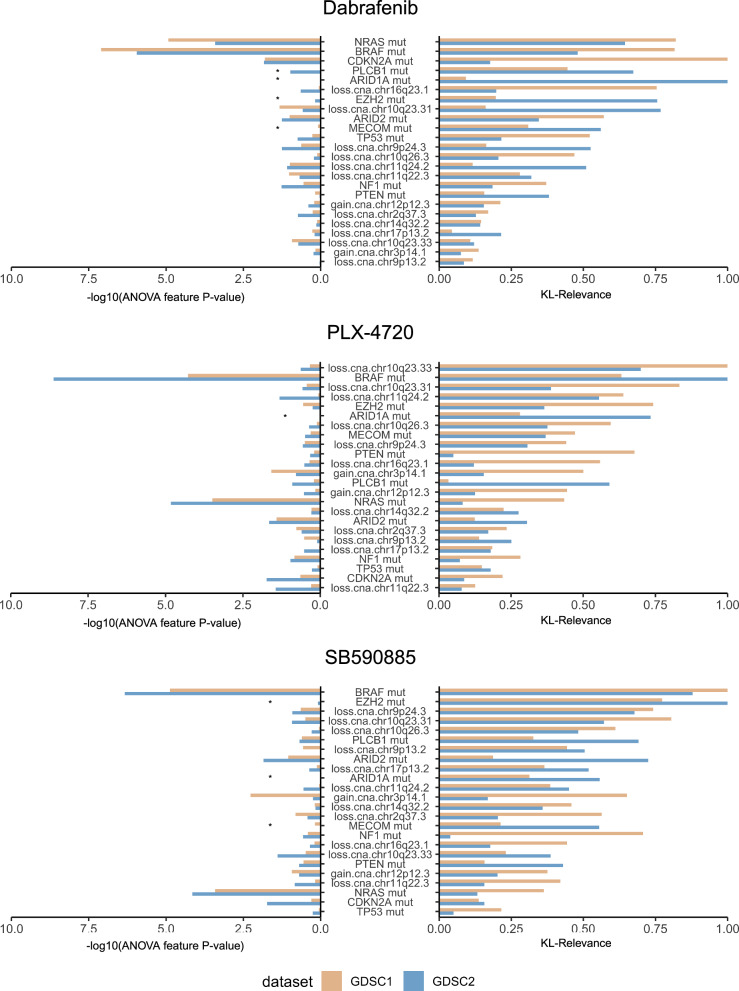
Melanoma-specific molecular features ranking using the KL-Relevance approach. Feature importance ranking, based on the average KL-Relevance scoring values between three drugs: Dabrafenib, PLX-4720 and SB590885, were computed using drug responses from GDSC1 and GDSC2, and compared with ANOVA test associated *p*-values for the same features. The number of cell lines (N) treated with each drug used in this dose–response dataset of SKCM is as follows: Dabrafenib (GDSC1: *N* = 39 ;GDSC2: *N* = 47), PLX-4720 (GDSC1: *N* = 40; GDSC2: *N* = 50) and SB590885 (GDSC1: *N* = 35; GDSC2: *N* = 45). The asterisk (*) denotes melanoma-specific features that were omitted from the ANOVA analysis of the GDSC1 or GDSC2 dataset because they were not present in a minimum of 3 cell lines. Cancer features are sorted according to the average KL-Relevance values obtained from both GDSC1 (trained on GDSC1) and GDSC2 (trained on GDSC2) datasets, presented in descending order. The greatest score represents the cancer feature of highest importance, as identified by the KL-Relevance approach, in modulating cell responses to specific drug treatments.

In the first experiment, we compared the importance of genomic features in the MOGP models for describing dose–response, as determined by the KL-Relevance, with features ranked by the feature *p* values derived from the ANOVA analysis conducted using a linear model, as reported in the GDSC database. This analysis was performed using the responses of three different drugs targeting BRAF: i) Dabrafenib, ii) PLX-4720 and iii) SB590885, and the same set of melanoma-specific features used in the ANOVA analysis. ANOVA was employed to assess the association between cancer features and drug responses, using the IC50 metric. A total of 24 cancer features were ranked based on the effect they have on the model’s prediction using the computed average KL-Relevance scoring values, compared with the resulting *p*-values obtained from the ANOVA analysis of both GDSC1 and GDSC2 dataset (Fig. 3).

Among the 24 cancer features frequently found in melanoma cell lines, *BRAF* mutation was shown to be the most important genomic feature for SB590885 dose–response, and second for drug Dabrafenib and PLX-4720 using the KL-Relevance approach. The top ranked feature, *BRAF* mutation, along with the mutation of its upstream activator in the same signalling pathway, *NRAS*, were consistently among the most statistically significant features associated with the responses to all three *BRAF* inhibitors by ANOVA analysis. This observation is correlated to the fact that all of these three drugs are designed to inhibit *BRAF* mutants. However, the highly ranked feature of *NRAS* mutation by ANOVA was only being detected as the most important feature for Dabrafenib drug responses prediction, but not PLX-4720 and SB590885 in KL-Relevance. Similarly, *CDKN2A* mutation was found to have less impact on both PLX-4720 and SB590885 response prediction. Furthermore, the loss of chr9p24.3 region, where the *CDKN2A* gene is located, emerged as an important feature for predicting SB590885 responses.

**Fig. 4:**
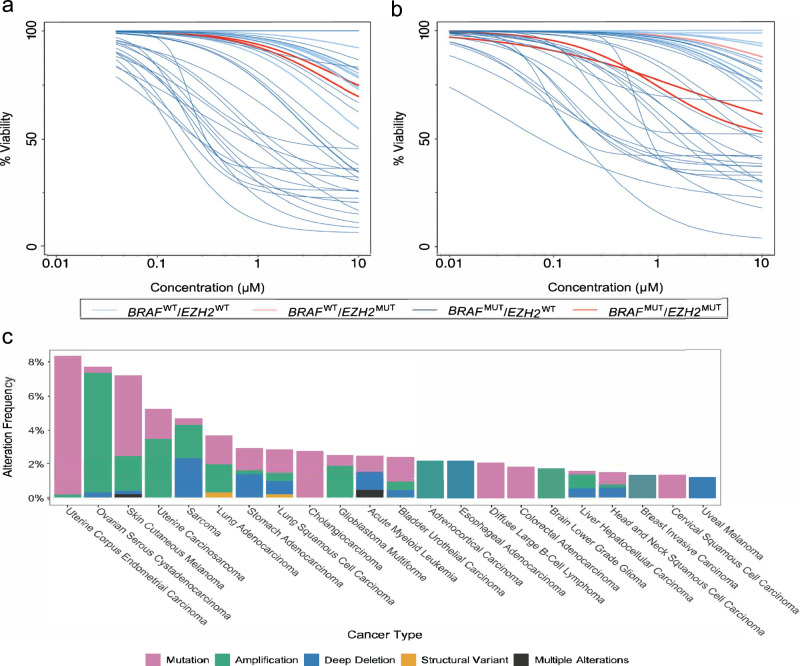
Differential drug responses in melanoma cell lines with distinct BRAF and EZH2 mutational status. Dose response curves of cell lines treated with PLX-4720, categorised by BRAF and EZH2 mutation status as follows: i) BRAF^WT^/EZH2^WT^ (*N* = 8), ii) BRAF^WT^/EZH2^MUT^ (*N* = 1), iii) BRAF^MUT^/EZH2^WT^ (*N* = 27), iv) BRAF^MUT^/EZH2^MUT^ (*N* = 2), obtained from **a** GDSC1 (*N* = 37) and **b** GDSC2 (*N* = 38) screening. A total of 37 melanoma cell lines were tested with PLX-4720 in both GDSC1 and GDSC2, except for cell line COSMIC ID: 909726 (BRAF^WT^/EZH2^MUT^), which was only in GDSC2. **c** The top 22 human cancer types with frequent genetic alterations in the *EZH2* gene. The *EZH2* gene exhibited alterations in 7.2% (mutation: 4.7%) of the 444 melanoma cases in TCGA PanCancer Atlas dataset, obtained from cBioPortal^58–60^ (accessed September 2023).

Interestingly, the *EZH2* gene mutation emerged as the second-highest ranked feature in the KL-Relevance model for drug SB590885. This feature also appeared as the fifth and seventh most important feature in the case of PLX-4720 and Dabrafenib, respectively. However, it was found to be the least or not significantly associated with differential responses to all three drugs in the ANOVA analysis. To further evaluate the impact of this novel predictive biomarker identified by KL-Relevance approach, we then assessed drug responses in four melanoma cell lines under varying *EZH2* and *BRAF* statuses treated with PLX-4720 (Fig. 4a, b). It was evident that the treatment of the PLX-4720 drug yielded different response outcomes across the four distinct melanoma cell lines. Treatment of drug PLX-4720 in cell lines with *BRAF* mutation (BRAF^MUT^) were more sensitive compared to *BRAF* wild-type (BRAF^WT^) cell lines, as expected for its use in BRAF-targeted therapies. In addition, *BRAF* mutant cell lines with *EZH2* mutation (EZH2^MUT^) were found to be less responsive to PLX-4720 than *EZH2* wild-type (EZH2^WT^) cell lines. The presence of *EZH2* somatic mutations has been observed in a subset of melanoma cases, as shown in The Cancer Genome Atlas (TCGA) dataset (Fig. 4c). The *EZH2* mutational status may indeed impact sensitivity to BRAF inhibition, providing a potential explanation for the development of resistance to BRAF inhibition in certain melanoma cases.

Although the KL-Relevance determination method is typically computed on the same dataset used for MOGP model training, we have also performed reverse tests by using the MOGP model trained on GDSC1 to determine KL-Relevance on the GDSC2 test dataset and vice versa, aiming to evaluate the method’s robustness across different drug screening studies. Supplementary Fig. 2 shows the feature rankings obtained from these tests consistently highlighted *BRAF* and *EZH2* mutations, as well as loss of chr10q23.33 region, as important features in predicting the dose–response of drug PLX-4720.

To further broaden our comparative results, we also included features ranking using the Shapley additive explanations approach (SHAP)^30^. Supplementary Fig. 3 shows a ranking comparison between the ANOVA, our KL-Relevance and SHAP drawn from the same set of melanoma-specific features used in the ANOVA analysis from the GDSC2 dataset tested with drugs Dabrafenib, PLX-4720 and SB590885. It is evident that there is a notable difference between the ranking values obtained by the KL-Relevance method compared to ANOVA and SHAP, regarding the weights assigned to the features. The KL-Relevance method generally assigns relevance values that decrease gradually from the most relevant to the least relevant. On the contrary, ANOVA and SHAP tend to rank a few features with high values and aggressively weigh the others with relatively small relevance values. For drugs PLX-4720 and SB509885, there is a good consensus between KL-Relevance and ANOVA in assigning high relevance to the BRAF mutation feature. However, this is not the case for drug Dabrafenib, where the BRAF mutation was not ranked in the top three, even though KL-Relevance considers this feature moderately important. Both KL-Relevance and SHAP considered the loss.cna.chr9p24.3 feature as relevant, but SHAP assigned this feature a higher ranking value than KL-Relevance. All three methods, KL-Relevance, ANOVA and SHAP, highlighted the significance of the BRAF mutation feature for drug PLX-4720. On the other hand, features such as NRAS mutation, gain.cna.chr3p14.1 and TP53 mutation showed inconsistent rankings across the methods. It is also worth noting that the SHAP method relies partly on the predictions from the MOGP model since it is not taking into account the (co)variance uncertainty measure provided by the MOGP. To highlight, the EZH2 mutation consistently emerged as one of the relevant features identified by the KL-Relevance method.

### Prediction of dose–response is robust between screening studies

**Fig. 5:**
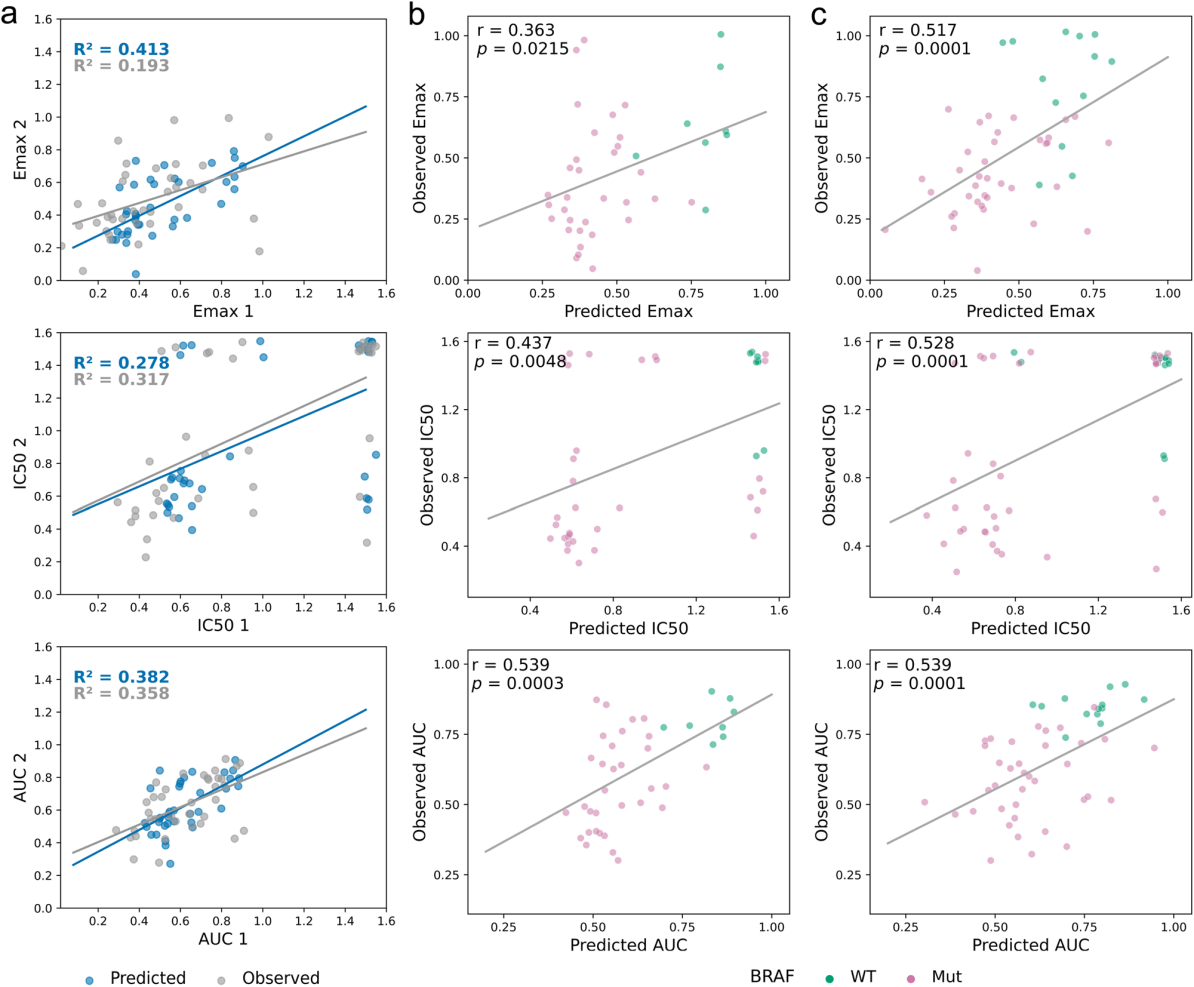
Evaluation of the MOGP model predictive performance in melanoma using three drug response metrics: Emax, IC50, and AUC. **a** Each dot in the figures represents a specific melanoma cell line treated with drug PLX-4720 in GDSC1 (1) (*N* = 37) versus GDSC2 (2) (*N* = 37). These metric values were obtained from predicted (blue) and observed (grey) dose–response curves of melanoma cell lines treated with drug PLX-4720 common between GDSC1 and GDSC2. Coefficient of determination, *R*^*2*^, was used to evaluate the variability levels of the drug responses of the common cell line across GDSC1 and GDSC2 using the computed dose–response metrics. Evaluation of the predictive performance of MOGP in predicting dose–responses of numerous melanoma cell lines in **b** GDSC1 (*N* = 40; WT = 8, Mut=32) and **c** GDSC2 (*N* = 50; WT = 13, Mut = 37) treated with drug PLX-4720, in relation to BRAF mutation status (wild-type: green; mutation: pink). The values of three different drug response metrics: i) Emax, ii) IC50, and iii) AUC were obtained from predicted dose–responses and compared with values derived from observed curves. The predictive power of the MOGP model was measured using Pearson correlation coefficient, *r*, to compare the values of MOGP-predicted and observed dose–response metrics.

Next we tested the ability of the MOGP models to predict dose–response data using genomic and drug chemistry features. We trained two MOGP models using dose response data of SKCM cell lines treated with PLX-4720: one trained with GDSC2 and tested on GDSC1, while the other used GDSC1 as the training dataset with GDSC2 as the testing dataset. GDSC1 comprises 40 melanoma cell lines, while GDSC2 consists of 50 melanoma cell lines, with 37 cell lines common to both datasets. Drug response metrics such as i) Emax, ii) IC50, and iii) AUC were derived from the dose–response curves (DRCs) predicted using the MOGP model. The values of these three metrics were also computed from the DRCs fitted using the sigmoid function with four parameters, and were then compared with the values obtained from the MOGP-predicted dose–response curves. We compared summary metrics of Emax, IC50 and AUC for the cell lines common between GDSC1 and GDSC2 (Fig. 5a). By using these three metrics, we assessed the variability in the dose–responses for identical cell lines treated with PLX-4720 between GDSC1 and GDSC2. Emax values obtained from the MOGP-predicted dose–response curves have the highest *R*^2^ score of 0.41, followed by AUC with an *R*^2^ score of 0.38, and the lowest *R*^2^ score of 0.28 for IC50. Whilst the *R*^2^ score achieved on IC50 and AUC was 0.32 and 0.36 respectively on values derived from observed DRCs, the *R*^2^ on Emax was only around 2. Overall, the *R*^2^ scores for all metrics, whether from observed or predicted dose–response curves, remained consistently in the range of 0.3–0.4. This observation strongly implies the existence of variations in dose–responses between GDSC1 and GDSC2, even when they are the same cell line-drug pairs.

We then further evaluated the predictive performance of the MOGP model by computing Pearson correlation coefficients between metric values derived from predicted and observed DRCs across all cell lines common between GDSC1 and GDSC2, and also those unique to either GDSC1 and GDSC2 (Fig. 5b, c). Of all three drug response metrics, AUC provided the most consistent and highest predictive performance with the best correlation of 0.54 in both GDSC1 and GDSC2. Generally, most cell line pairs demonstrated good correlations between Emax, IC50, and AUC values derived from predicted dose–responses and those from observed curves in GDSC2, with correlation coefficients exceeding 0.50. In GDSC1, on the other hand, IC50 showed a correlation of 0.44 while for Emax was merely 0.36. These correlations indicated that the prediction of dose–responses in GDSC2 performed marginally better than that in GDSC1, particularly for metrics such as Emax and IC50. However, the differences we observed here in Emax and IC50 could be attributed to the noises arising from the variability in drug responses between the common cell line pairs in GDSC1 and GDSC2 (Fig. 5a). Importantly, these potential technical noises appeared to have minimal, if any, impact on the accuracy of our model’s predictions using the AUC metric. The predictive performance of our model on dose-–response in a small number of cell lines present only in GDSC1 or GDSC2 was suboptimal, showing weak to moderate correlations that were not statistically significant (Supplementary Fig. 4).

### Drug response prediction across cancer types with varying training set sizes

**Fig. 6:**
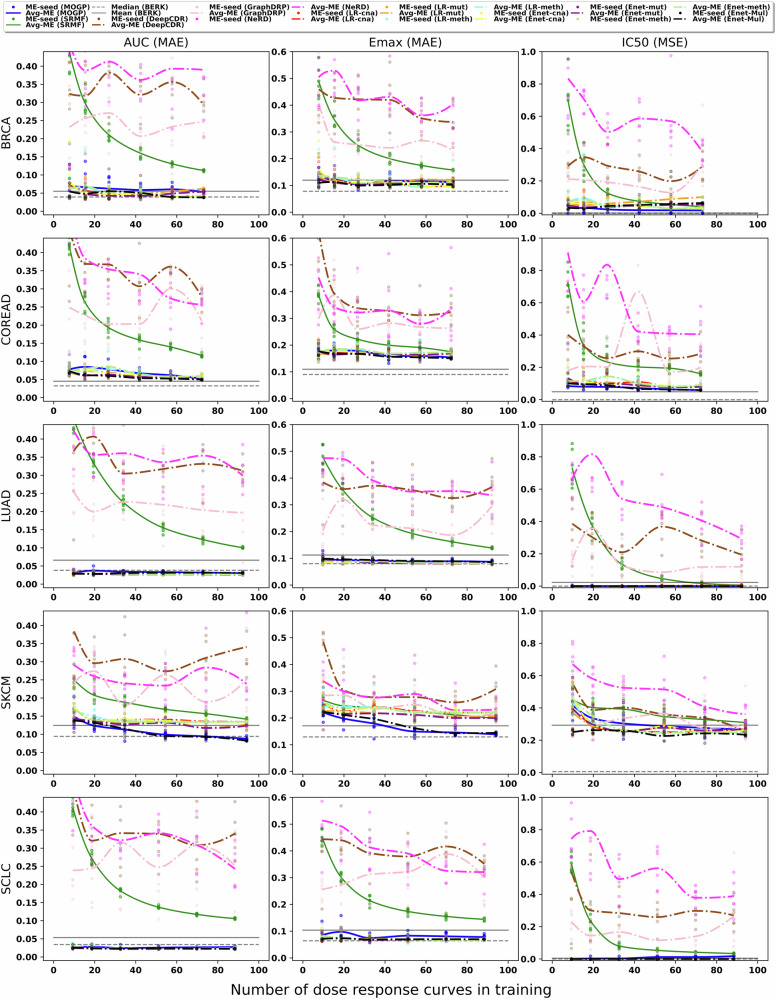
Performance of predictive methods. MOGP, SRMF, DeepCDR, GraphDRP, NeRD, Lasso Regression (LR) and Elastic Net (Enet) were assessed with respect to the summary metrics AUC, Emax and IC50 when increasing the number of DRCs in training. Training and prediction was performed on each cancer type separately. From left to right: BRCA $(N=110)$, COREAD $(N=108)$, LUAD $(N=139)$, SKCM $(N=142)$ and SCLC $(N=133)$; 30% of each cancer dataset is used as testing. Each subfigure shows dots aligned vertically that correspond to the Mean Error of the methods per seed; the dots correspond to the six different random seeds used to sample each training set that was gradually incrementing in size. The gradual increment of the DRCs for training the model is as follows: {8,16,27,42,58,74} for BRCA, {7,15,26,42,57,72} for COREAD, {10,19,34,53,72,91} for LUAD, {10,20,35,54,74,93} for SKCM and {9,18,32,51,69,87} for SCLC. Each increment of curves in training yields six Mean Errors as per the six seeds, thus, along the number of DRCs in training, the solid line interpolates the averages of Mean Errors between the seeds at each increment. A strong benchmark (BERK) error metric for each of the summary metrics is obtained as the error between GDSC1 and GDSC2 screens for the same drugs and cell lines; we report the mean and median BERK error. LR-mut Lasso regression with mutation profiles, LR-meth Lasso regression with methylation profiles, LR-cna Lasso regression with copy number alterations, Enet-mul Multi-task learning Elastic Net with all the omics data and drug features.

Given the challenges in obtaining samples from certain cancer types and the substantial costs involved in drug screening experiments, our second experiment was designed to evaluate the predictive capabilities of the MOGP model across five distinct cancer types. We examined how the model’s prediction performance varied with different numbers of DRCs for training, and subsequently reported the performance over the test set. We used six different random seeds to sample each training set that was gradually incrementing in size. The error in the MOGP’s predictions was computed when the number of DRCs in training is varied at each of the seven dose concentrations (Supplementary Fig. 5) and across all doses when evaluating summary metrics AUC, Emax and IC50 (Fig. 6).

Increasing the number of DRCs in training, our model’s prediction performance improves for the BRCA, COREAD and SKCM cancers; by averaging along doses there is a reduction in their absolute error from the smallest to largest number of DRCs in training of 0.029 ± 0.010, 0.025 ± 0.011 and 0.073 ± 0.015, respectively. On the other hand, the LUAD and SCLC cancers do not improve much, presenting almost a flatten behaviour whilst increasing the DRCs in training.

We have benchmarked our MOGP with 3 DL methods (DeepCDR^17^, GraphDRP^18^, and NeRD^16^) and 3 non-DL based ML methods (SRMF^23^, Lasso Regression, and Elastic Net^31^). The previous methods had previously been trained and tested across all cell lines (pan-cancer), but here we conduct the experiment in each cancer type separately. The detailed information of these models is introduced in Supplementary Table 1 and Methods Section: Benchmark Methods. Among the DL models, GraphDRP, which uniquely relies on the mutation data, demonstrated the most favourable results within its category but still lagged behind MOGP and the ML models in overall efficacy. With the largest number of DRCs, GraphDRP achieves AUC average-MAE values between 0.2 and 0.25, while NeRD, integrating multi-omics data, shows AUC average-MAEs between 0.25 and 0.35. DeepCDR records the most unfavourable performance, with AUC average-MAE scores greater than 0.3 across all cancer types studied.

On the other hand, non-DL methods show robust prediction performance, with SRMF and MOGP methods improving accuracy as the number of DRCs increases. Nonetheless, when trained with fewer than 25 DRCs, MOGP significantly outperforms SRMF in prediction error across many cancer types. For instance, MOGP’s average-MAE for AUC ranges between 0.02 and 0.15, while SRMF ranges between 0.2 and 0.42. Similarly, MOGP’s average-MSE for IC50 ranges between 0.001 and 0.41, whereas SRMF’s ranges between 0.1 and 0.75. Since BRAF inhibitors are more effective in SKCM, we measured prediction error in this cancer split by the drug non-responsive and responsive cases. Supplementary Fig. 6 shows that for both cases the MOGP model reached a salient performance close to BERK, whereas SRMF struggled.

### Scaling up analysis to multiple cancer types and a broader range of drugs targeting different biological pathways

To evaluate the performance of our MOGP model in predicting dose–response curves of a diverse range of drug compounds across cell lines representing multiple cancer types, as commonly observed in drug screening studies, we expanded our analysis to include additional training and testing on independent data sourced from GDSC2 datasets. In addition to the five cancer types used in our previous experiments above (SKCM: *N* = 456, BRCA: *N* = 441, COREAD: *N* = 429, LUAD: *N* = 537 and SCLC: *N* = 501), this analysis also included dose responses of five additional cancer types: pancreatic adenocarcinoma (PAAD): *N* = 253, ovarian serous cystadenocarcinoma (OV): *N* = 292, head and neck squamous cell carcinoma (HNSC): *N* = 305, acute lymphoblastic leukaemia (ALL): *N* = 244, and oesophageal carcinoma: *N* = 319. We divided the drug response data for each cancer type into 70% and 30% for training and testing our MOGP model, respectively. These dose responses were obtained from the treatment of ten different drugs: Vorinostat, Axitinib, PLX-4720, MK-2206, Pictilisib, AZD8055, SB590885, TW 37, Trametinib and Dabrafenib, targeting different biological pathways, including ERK/MAPK signalling, PI3K/MTOR signalling, RTK signalling, apoptosis regulation and chromatin histone acetylation. It is worth mentioning that we split the datasets by ensuring that 70% of DRCs per drug is present in the training set and 30% of DRCs per drug is present in the testing set; i.e., if there are 10 DRCs for drug dabrafenib and 20 DRCs for drug PLX-4720 in the dataset, then we guarantee that there will be 7 and 14 DRCs for training, and 3 and 6 DRCs for testing from dabrafenib and PLX-4720, respectively. By means of a cross-validation process, we trained 44 models for each cancer type. The training of each model was implemented using an ADAM optimiser in a High Performance Computing system with 32 CPU cores and 24 GB of memory.

Supplementary Figs. 7–11 show the performance of the MOGP model over the test sets of each cancer regarding the Emax, IC50 and AUC summary metrics. The results demonstrate across the ten cancer types that the Emax is the metric with the highest Pearson correlation performance with average and standard deviation of 0.783 ± 0.069, followed by AUC with 0.717 ± 0.051 and IC50 with 0.653 ± 0.125. We further assessed the performance of our MOGP model in predicting the dose response curves for drugs targeting various pathways. In Supplementary Fig. 12a, COREAD cell-drug pairs targeting the ERK/MAPK or PI3K/MTOR pathway showed robust correlations between the predicted and observed values of Emax, IC50 and AUC, with correlation coefficients nearing 0.7. Similarly, in another cancer type SCLS, we observed strong correlations (*r* ≥ 0.6)between predicted and observed metric values for similar drugs targeting the PI3K/MTOR pathway (Supplementary Fig. 12b), but not for those drugs targeting the ERK/MAPK signalling pathway. This limitation could arise due to the limited availability of sensitive response data from the treatment of ERK/MAPK-targeted drugs on these SCLC cell lines in the training dataset, potentially impacting the predictive power of these drugs’ response curves on these cell lines (Supplementary Fig. 13).

## Discussion

Our study shows that the MOGP approach was able to rank the impact of genomic features on melanoma cell lines’ responses to three BRAF inhibitors, identifying *BRAF* and *NRAS* mutations as consistently critical predictors while also highlighting the previously underappreciated role of *EZH2* mutations that might offer insights into differing drug sensitivities and potential resistance pathways in melanoma therapies. MOGP models were trained on the GDSC1 and GDSC2 datasets separately to predict drug responses (Emax, IC50, AUC) that were substantially correlated with observed responses, *R*² scores ranging between 0.3 and 0.4. They demonstrated a slightly better predictive performance in GDSC2, particularly for Emax and IC50 metrics, while maintaining that AUC remains the most robust metric for predicting dose–responses across both datasets, showing the least susceptibility to potential technical noises. MOGP model’s performance in predicting drug responses was also robust and we did not see a significant decrease in performance using smaller training sets (as low as 9 cell lines) of dose–response curves in different cancer types. This was evidenced when assessing response at each individual dose and when comparing to other benchmarks.

The discovery of the genomic feature related to *EZH2* through the KL-Relevance approach highlights its impact on drug responses and potential involvement in drug resistance in cancer cells. *EZH2*, known for silencing critical tumour suppressor genes^32,33^, has been implicated in BRAF-targeted therapy resistance in melanoma^34,35^. Modulation of *EZH2* has shown promising results in addressing resistance to BRAF inhibitors^35^. Furthermore, our findings have also highlighted the distinct genomic feature effects on responses to three different BRAF inhibitors, likely dependent on their respective biochemical mechanism and their conformation in which they stabilise the RAF kinase^36,37^. Dabrafenib exhibits greater efficacy in suppressing the growth of cancer cell lines with *NRAS* mutations compared to Vemurafenib (its analogue, PLX-4720), which aligns with our KL relevance ranking^38–40^. Treatment with PLX-4720/Vemurafenib or SB590885 have been shown to enhance MAPK activation, even in wild-type *BRAF* expressing cells, through RAF dimerisation and transactivation^38,41,42^. Consequently, the impact of *CDKN2A* mutation on the efficacy of PLX-4720/SB590885 treatment appears to be less significant in this context, considering dysregulation of cell-cycle signalling through the upstream MAPK pathway activation. Our method has also identified the loss of the chr9p24.3 region, containing both *CDKN2A/B* genes responsible for cell cycle regulation, as a significant feature associated with drug sensitivity of SB590885. This observation aligns with previous findings on disruptions in the regulatory components of the cell cycle progression pathway, contributing to SB590885 resistance in *BRAF* V600-mutated melanoma^43^. Our KL-Relevance method exhibited modest enhancement over ANOVA or SHAP in identifying significant genomic features contributing to differential drug responses with specific drugs, uncovering insights not previously detected by ANOVA.

Modelling the complete curve response by means of a MOGP model might become prohibitive in the context of large numbers of drugs and cell lines. This is due to having a computational complexity for a regression task of $O({N}^{3}{D}^{3})$, where $N$ refers to the number of data observations, i.e., dose responses in our case; and $D$ represents the number of outputs, i.e., the number of drug concentrations in our datasets with $D=9$ for GDSC1 and $D=7$ for GDSC2 datasets. Although, there exist sparse MOGP models^44,45^ that allow scalability when $N$ is large by means of approximations of the exact MOGP model, they present a limited performance when applied to scenarios where the input features are much bigger than hundreds^46^. Thus, investigating the applicability of sparse approaches for massive genomic and drug compounds features could be a challenging venue for future research. We preferred to avoid such sparse approximations and opted for the exact MOGP model to explore how accurately it would perform; anyhow, its application is a feasible choice in a context of hundreds or even thousands of dose responses. Supplementary Fig. 14 shows an example of the time distribution for training the MOGP model to predict the DRCs of ten different cancer datasets that present different $N$ sizes. On the other hand, we noticed that most of the DRCs behave as non-responsive with respect to the drugs causing an unbalanced distribution along the dose concentrations. Therefore, though we have achieved relevant results by applying our MOGP model, using a different type of likelihood in Eq. (1) that accounts for the data unbalanced behaviours might help to improve the predictions^11,26^. Apart from the experiments presented in this main manuscript, we investigated the performance of the MOGP model to predict the response curve of a cancer A that emulates a rare cancer with a very small number of DRCs available for training (Supplementary Fig. 15). We wanted to understand if the data information known from common cancers could be transferred to improve predictions on a rare cancer, i.e. if the predictive performance of our MOGP model that targets the dose–response of a cancer A (with very few observations) could be improved by means of feeding the model with dose–responses from cancers different to such a cancer A. We found that increasing the training dose–responses from other cancers overfits the model, so the training error improves while the testing error over cancer A degrades. We believe that the current modelling structure of the MOGP model should be rebuilt in order to deal with this type of rare cancer scenario. For instance, works like^47^ and^48^ introduce feasible ideas for transferring learning between source domains of data (like the common cancers in our application) to improve the predictive performance on a specific target domain that lacks data (like the rare cancer A in our experimental case).

Although DL methods have been studied to show stronger performances against conventional ML methods in large scale drug response datasets^9^, they exhibited inferior performance compared to both MOGP and the selected ML algorithms within the constraints of limited training data (less than 100 training samples). Our analysis revealed that the integration of multi-omics genomic features by NeRD and DeepCDR did not inherently enhance model performance in the context of training and predicting within a singular cancer type. This outcome can be attributed to the inherent complexity of DL models. Despite the strategic implementation of an early-stopping criterion on validation set performance to prevent overfitting and facilitate optimal convergence, DL models struggled to achieve a better behaviour than non-DL models in a limited training data scenario. A challenging setting in the benchmarking process was the inclusion of only three drugs, constraining the capacity of drug encoders to adequately learn and predict drug responses. It is important to recall that DL models are generally fed with large amounts of data to achieve a salient performance; nonetheless, in regimes of few data observations they are prone to poorly generalise. As shown in our experiments, simpler models like Elastic-net and Lasso regression can even reach better error metrics than models with more complex architectures. It is worth emphasising that most cell lines are non-responsive to the drugs tested, except in SKCM cell lines, indicating that the IC50 may exist beyond the maximum concentration tested. We set a hard maximum of IC50 = 1.5 in those cases, which may introduce bias into the data and distort performance evaluation. The alternative is to extrapolate the dose–response curves beyond the maximum concentration, but it has been previously shown that there is too much uncertainty in these extrapolate IC50 estimates to be used for drug assessment or biomarker detection^11^. Although the MOGP did not always achieve the highest performance in all the cases, it stands out by its training on observed drug responses at varied doses but not on specific summary metrics. Unlike other models that treat IC50, Emax, and AUC as separate tasks and require individual training sessions for each metric, MOGP concurrently predicts these drug response summary metrics within a unified framework and achieves competitive results.

Given our understanding of the uncertainty associated with measuring drug response and molecular features, it is crucial for models that predict drug response to provide full dose–response curves and probability estimates, not just summary point estimates. Utilising probabilistic multi-output models presents a forward-leaning approach in precision medicine, offering a nuanced understanding of drug efficacy across various dose metrics. This approach is notably beneficial when the available training data is scant, as it enables the capture of underlying response trends across all doses, fostering a granular understanding of drug efficacy. By encompassing a comprehensive dose spectrum, researchers can meticulously decipher the varying response dynamics exhibited at distinct dose levels, potentially unveiling novel insights into the drugs’ mechanism of action. Importantly, it sidesteps the limitations seen in models exclusively trained on summary metrics like IC50, Emax, or AUC, by offering insights across a continuum of dose–responses, thus potentially uncovering intricate response patterns and facilitating a more comprehensive understanding of the pharmacodynamics involved. This approach harbours significant promise in enhancing drug development pipelines by economising on data requirements while simultaneously elevating the precision in predicting dose–response relationships.

## Methods

### Dose–response

Drug response data were extracted from the Genomics of Drug Sensitivity in Cancer (GDSC), which contained two pharmacogenomic screens; GDSC1 and GDSC2. GDSC1 includes data from 2010 to 2015 whilst GDSC2 is newer and includes screens conducted post-2015. Hundreds of compounds have been screened across approximately 1000 cell-lines with two key studies published using GDSC1. We used version 8.4 of the datasets released July 2022 in this study. In short, 403 compounds have been screened across 970 cell-lines to produce 333292 IC50s in GDSC1 and 297 compounds were screened across 969 cell-lines giving 243,466 IC50s in GDSC2.The two datasets also use different assays for screening with GDSC1 using Syto60 (Resazurin) and GDSC2 using CellTitreGlo. The duration for both these assays is 72 h.

In the GDSC1 dataset, dose–response data was generated through drug screening with two different concentration protocols: one involving 9-dose (2-fold dilutions) and the other with 5-dose (4-fold dilutions) concentrations. For the dataset used in our study, only dose responses of SKCM cell lines treated with three BRAF-targeted drugs (Dabrafenib, PLX-4720 and SB590885) were included, primarily with 9-dose concentrations. If a drug was screened at both 5- and 9-dose concentrations, dose responses with 5-dose concentrations were excluded. Also, dose responses in GDSC1 were excluded from the dose-specific drug responses prediction experiment in ten different cancer types. In contrast, GDSC2, which was used specifically for the dose-specific drug responses prediction in these ten cancer types, utilised a 7-dose concentration protocol. This protocol included a 7-point dose curve with a half-log dilution step covering a 1000-fold range and another 7-point dose curve with 2 × 2-fold dilutions followed by 4 × 4-fold dilutions, covering a 1024-fold range. However, only the 7-point dose curves with the 1000-fold range drug treatment is included in our study. The raw screening data from both GDSC1 and GDSC2 were processed using the R package, *gdscIC50*. This R package was used to process raw data in the GDSC database and fit dose–response curves for obtaining the AUC and IC50 values presented on the GDSC website (http://www.cancerrxgene.org). In our study, this R package was only used for several data pre-processing steps: data filtering, viability data normalisation, and normalisation of the drug treatment concentration scale. Briefly, screening data with any missing drug information or having failed internal quality control processes indicated by a “FAIL” tag, were first removed. The remaining raw viability data were then normalised with the negative (viable cells with media in GDSC1 or media and compound vehicle in GDSC2) and positive (no viable cells) controls of each tested plate, identified using tags (e.g. neg_control = “NC-0” or “NC-1”, pos_control = “B”) present in the dataset. Distinct drug treatment concentration ranges used in the screening of different individual drugs were then normalised on a single scale in each dataset to make them comparable across all drug concentrations used.

### Drug chemistry

In addition to the genomic features, a total of 27 chemical features relating to drug molecules such as molecular weight, hydrogen bond donor and acceptor counts and formal charge were initially used. These were extracted from PubChem using PubchemPy and the definitions for each feature can be found under “Computed Properties” for any compound on PubChem. Additionally, the presence and absence of elements in each compound has been encoded and used as binary features. These include boron, nitrogen, phosphorous, platinum, iodine, chlorine, oxygen, sulphur, bromine and fluorine. The final set of chemical features comprises 12 features, as shown in Supplementary Table 2.

These chemical features or molecular descriptors can vary in dimensionality (1D, 2D, 3D and so on) and quantitatively illustrate molecular properties of a chemical compound^49^. For the purposes of this project, only 2D descriptors were used alongside encoded elements. These 2D descriptors represent information gathered from the structural formula of the compound and include many subdivisions such as topological indices and simple counts^50^. The latter, as the name suggests, refers to features in a compound that can be described by simple counts such as hydrogen bond donors and molecular weight. The former refers to descriptors derived from 2D graphs of the molecules or compounds and includes features such as complexity.

### Molecular data

The status of three molecular features: i) genetic variations in high confidence cancer genes, ii) CNA status of RACSs and iii) DNA methylation status of iCpGs for cancer cell lines in the GDSC database were identified using cancer functional events (CFEs) found in the primary tumour samples by combining data across all tumours (pan-cancer) or for each specific cancer type.

For comparative analysis between ANOVA and KL-relevance: i) analysis of variance (ANOVA) and ii) KL-Relevance used to determine the contributions of specific cancer features to the variability in drug responses in skin cutaneous melanoma cell lines, a multi-omics binary event matrix (MOBEM) containing the status of a total of 24 melanoma-specific CFEs (genetic variations and CNAs) detected in the associated cell lines were extracted, which were used as input for ANOVA statistical analysis to determine associations between cancer features and drug sensitivity in the GDSC database (Supplementary Data 1). Among the complete set of 24 melanoma-specific CFEs, only those that occurred in at least 3 cell lines in GDSC1 or GDSC2 were taken into account for the ANOVA analysis. Details can be found in the Data Availability section.

For dose-specific prediction of drug responses across different cancer cell lines representing 10 cancer types, genomic annotation data of a panel of 1001 human cancer cell lines were obtained from the GDSC1000 resource site https://www.cancerrxgene.org/gdsc1000/GDSC1000_WebResources///Data/BEMs/CellLines/CellLines_Mo_BEMs.zip (Supplementary Data 2). The set of pan-cancer CFEs in a binary event matrix consisted of 1073 molecular features in total (genomic variants: *N* = 310, CNAs: *N* = 425, DNA methylation: *N* = 338). Briefly, genetic variants, CNAs and DNA methylation status of the selected cancer cell lines were identified as described^31^. Individual genetic variants found within a coding region of a set of 470 high confidence cancer genes, based on their frequent occurrence in COSMIC v68 (http://cancer.sanger.ac.uk/cosmic/), were identified. The mutational status of these genetic features in the cancer cell lines were then determined. For CNAs, focal amplification/deletion of chromosomal regions were identified for all cell lines in the panel. Only amplified regions spanning at least a gene or deletion events of an exon were included. Identification of iCpGs, regions where beta signals are distributed bimodally, was carried out in each specific cancer type separately due to the highly tissue specificity of the DNA methylation profiles. DNA methylation status of these iCpGs were then determined and only hypermethylated sites in the gene promoter region were included. The presence or absence of these different features in the selected cell lines were encoded with binary values. These binary features of cell lines were then used in the MOGP modelling for drug response prediction. The cancer features of the 279 cancer cell lines were first extracted from this set of pan-cancer CFEs for use in the aforementioned analysis (Supplementary Data 3). Similar dose–response predictions were further expanded to include an additional 436 cell lines from the GDSC2 dataset representing additional cancer types (*N* = 10), and a wider range of drug compounds (*N* = 10) targeting 5 biological pathways. The molecular features of the additional cell lines used in the analysis are presented in Supplementary Data 4).

### Multi-output Gaussian process regression model

A Gaussian process (GP)^51^ is a distribution over functions generally used as a non-parametric prior for a probabilistic model. Here, we apply a probabilistic regression model based on GPs to predict the dose–response of different drugs applied to a particular type of cancer. We have a set of $N$ input data observations $X={[{x}_{1}^{T},...,{x}_{N}^{T}]}^{T}$, where each data observation ${x}_{n}$ is a multi-modal vector of $P$ features; they are a combination of genomics and drugs features. We apply the Multi-output GP (MOGP) model where each output is associated with a dose concentration^52^, i.e., there is a particular $d$-th output vector, ${y}_{d}={[{y}_{d,1},...,{y}_{d,N}]}^{T}$, that stacks $N$ data observations of the dose–response for the $d$-th drug concentration. We can express a joint distribution for a MOGP regression models as:

1 $$p(y,f{{|}}X)\,=\mathop{\prod}\limits_{d=1}^{D}\mathop{\prod}\limits_{n=1}^{N}G({y}_{d}{\rm{|}}{f}_{d}({x}_{n}),{\sigma }^{2})p(f),$$

where $G({y}_{d}|{f}_{d}({x}_{n}),{\sigma }^{2})$ is a Gaussian distribution with mean ${f}_{d}({x}_{n})$ and variance ${\sigma }^{2}$; $f={[{f}_{1}^{T},...,{f}_{D}^{T}]}^{T}$ is a vector built with ${f}_{d}={[{f}_{d}({x}_{1}),...,{f}_{d}({x}_{N})]}^{T}$, where each latent function is derived from an intrinsic coregionalization model^53^, ${f}_{d}(x)={\sum }_{i=1}^{R}{a}_{d,i}{u}^{i}(x)$, for which each ${u}^{i}$ is an independent and identically distributed (IID) sample taken from a GP, i.e., $u\sim {GP}(0,k(.,.))$, with $k(.,.)$ accounting for a kernel covariance; $R$ represents the number of IID samples taken from the GP; ${a}_{d,i}$ is a linear combination coefficient; and ${\sigma }^{2}$ is a noise parameter that aims to model the uncertainty of each dose–response ${y}_{d}$. The noise parameter ${\sigma }^{2}$ accounts for the noise variance of the dose–responses ${y}_{d}$. Such a noise parameter emerges from the construction of the regression model from a probabilistic perspective. We can think of the dose–response ${y}_{d}$ as an output generated by a function of the inputs $x$ and corrupted by noise: $y=f(x)+\epsilon$, where $\epsilon \sim N(0,{\sigma }^{2})$ is a Gaussian noise, with noise parameter ${\sigma }^{2}$, that corrupts the output $y$. Therefore, instead of modelling the outputs ${y}_{d}$ as a point estimate, it is more realistic to allow the output ${y}_{d}$ to be modelled with some uncertainty encoded in the parameter ${\sigma }^{2}$.

It is worth mentioning that the kernel covariance, $k(.,.)$, generally depends on a set of hyper-parameters, for instance an Exponentiated Quadratic (EQ) kernel, $k(x,x^{\prime} )={\sigma }_{{EQ}}^{2}\exp \left(-\frac{{|x}-x^{\prime} {|}^{2}}{{2l}^{2}}\right)$, relies on the length-scale $l$ and variance ${\sigma }_{{EQ}}^{2}$.

Training a MOGP regression model consists on maximising the log marginal likelihood:

2 $${\mathrm{log}}\,{\rm{p}}(y{\rm{|}}X)=-\frac{{ND}}{2}\log (2\pi )-\frac{1}{2}\log {\rm{|}}K+{\sigma }^{2}I{\rm{|}}-\frac{1}{2}{y}^{T}{(K+{\sigma }^{2}{I}_{N})}^{-1}y,$$

w.r.t to the kernel hyper-parameters, the coefficients ${a}_{d,i}$ and the noise parameter ${\sigma }^{2}$; here $y={[{y}_{1}^{T},...,{y}_{D}^{T}]}^{T}$ is the output vector that stacks all $D$ drug concentration outputs; ${I}_{N}$ is an identity matrix of size $N\times N$; and $K$ is a covariance matrix which entries are built with evaluations of the covariance, ${k}_{{MO}}(x,x^{\prime} )=\mathrm{cov}[{f}_{d}(x),{f}_{d^{\prime} }(x^{\prime} )]$, for all pairs of input data observations $X$, and all pairs of outputs $D$. In order to make predictions for a new set of ${N}_{* }$ input data observations ${X}_{* }$, we just need to evaluate the predictive distribution, ${G(y}_{* }|\mu ({X}_{* }),S({X}_{* }))$, with mean and covariance:

3 $$\mu ({X}_{* })={K}_{* }{(K+{\sigma }^{2}I)}^{-1}y,$$

4 $$S({X}_{* })={K}_{* * }-{K}_{* }{(K+{\sigma }^{2}{I}_{N})}^{-1}{K}_{* }^{T}+{\sigma }^{2}{I}_{* },$$

where ${K}_{* }$ is a matrix built with evaluations of the ${k}_{{MO}}(.,.)$ between the ${X}_{* }$ and $X$; ${K}_{* * }$ is a matrix built with evaluations of the ${k}_{{MO}}(.,.)$ between all pairs of observations in ${X}_{* }$; and ${I}_{* }$ is an identity matrix of size ${N}_{* }\times {N}_{* }$. The equations above provide us with a mean prediction $\mu$ and covariance matrix $S$ to quantify the uncertainty of our prediction.

### Kullback-Leibler relevance determination

In order to build a measure of predictive relevance of a $p$-th input feature is necessary to compute the MOGP predictive distribution from Eqs. (3) and (4) over a training data observations ${x}_{n}.$ Then, one compares how such a distribution differs when is recomputed with a subtle modification of the $p$-th feature of ${x}_{n}$. We can express the relevance of the $p$-th feature with respect to the data observation ${x}_{n}$ by calculating the divergence of the predictive distributions:

5 $$r(n,p,\varDelta )=\frac{d[{G(y}_{* }{\rm{|}}\mu ({x}_{n}),S({x}_{n})){\rm{||}}{G(y}_{* }{\rm{|}}\mu ({x}_{n}+{\varDelta }_{p}),S({x}_{n}{+\varDelta }_{p}))]}{\varDelta },$$

where $d[.{||}.]=\sqrt{2{D}_{{KL}}[.{||}.]},$ with ${D}_{{KL}}[.{||}.]$ as a Kullback-Leibler divergence, and ${\varDelta }_{p}$ as a vector of zeros with $\varDelta$ on the $p$-th entry. This kind of relevance determination has been previously studied in the context of single output Gaussian processes by^54^, here we extend the application of the relevance metric to the context of multiple-outputs, where ${y}_{* }$ represents a vector prediction of $D$ points of cell viability associated with the drug concentrations. Therefore, the divergence ${D}_{{KL}}[.{||}.]$ is computed between multivariate distributions with parameters $\mu \in {R}^{D}$ and $S\in {R}^{D\times D}$. Since we calculate the relevance per $n$-th datapoint, we can compute an average of the predictive relevance for a $p$-th feature along all the data observations,

6 $$K{L}_{p}=\frac{1}{N}\mathop{\sum}\limits_{n=1}^{N}r(n,p,\varDelta ),$$

and use this estimate to rank the $P$ features of the input data observations $X$ by their average relevance.

### Dataset for BRAF biomarker identification in melanoma

For all our experiments we selected three types of drugs that are highly correlated to the BRAF features: drug PLX-4720, SB590885 and Dabrafenib. There are two types of experiments: the first type mainly focuses on modelling the dose–response of melanoma cancer and identifies the relevance ranking of the features; we compare the ranking with the ANOVA method applied to the same data in the GDSC database for computing the degree of association between the genomic features (coding mutations and recurrent copy number alterations) of cancer cell lines and drug sensitivity measured by IC50 values. The second type of experiment aims to provide insights about the fundamental question of what is the threshold amount of cell lines or dose–response curves necessary for obtaining a robust predictive model for a particular type of cancer; we apply our model to BRCA, COREAD, LUAD, SKCM and SCLC cancers with dose–responses from drugs PLX-4720, SB590885 and Dabrafenib.

### Dataset for KL-relevance of BRAF drugs to compare with ANOVA

For the first experiment we use cell lines of melanoma cancer from both GDSC1 and GDSC2 datasets. The details are the following: GDSC1 has $N=40$ dose–responses for drug PLX-4720, $N=35$ for drug SB590885, and $N=39$ for drug Dabrafenib; the number of input features is $P=24$. These features are the same 24 input features used in the ANOVA analysis in the GDSC database, where only features found in at least three cell lines of a specific cancer (SKCM) were included in the analysis. GDSC2 has $N=50$ dose–responses for drug PLX-4720, $N=45$ for drug SB590885, and $N=47$ for drug Dabrafenib; the number of input features is also $P=24$ (also the same used in the ANOVA analysis). The number of drug concentrations is seven ($D=7$) in the GDSC2 dataset for all drugs, but in the GDSC1 dataset is nine ($D=9$) for drugs PLX-4720 and SB590885, and five $(D=5)$ for the drug Dabrafenib.

### Dataset for training, across cancer types and increasing size of training sets

For the second experiment we use cell lines of BRCA, COREAD, LUAD, SKCM and SCLC cancers from the GDSC2 dataset; their dose–responses are produced by the drugs PLX-4720, SB590885 and Dabrafenib. Thus, our dataset consists of BRCA ($N=110$), COREAD ($N=108$), LUAD ($N=139$), SKCM ($N=142$) and SCLC ($N=133$); we split each cancer dataset into 70% training and 30% testing, guaranteeing the number of drugs appearing in training and testing is proportional to the original number in the whole dataset. This dataset contains a total of 780 unique molecular features after filtering out those that have the same values across all dose–responses of specific drug-cell line pairs, which include 279 mutations, 418 CNAs, 71 related to DNA methylation and 12 chemical features of drug compounds. These $P=780$ input features are used to predict seven drug concentrations ($D=7$) that form the dose–response curve. In this second experiment, we examine how the MOGP model’s prediction performance varies with different numbers of DRCs for training and report the performance over the test set. The gradual increment of the DRCs for training the model is as follows: $\{\mathrm{8,16,27,42,58,74}\}$ for BRCA, $\{\mathrm{7,15,26,42,57,72}\}$ for COREAD, $\{\mathrm{10,19,34,53,72,91}\}$ for LUAD, $\{\mathrm{10,20,35,54,74,93}\}$ for SKCM and $\{\mathrm{9,18,32,51,69,87}\}$ for SCLC.

### Training of MOGP

In order to train the MOGP model we have to maximise the log marginal likelihood of Eq. (2) w.r.t the kernel’s length-scale hyper-parameter, the coefficients ${a}_{d,i}$ and the noise parameter ${\sigma }^{2}$. For the covariance kernel, when having a variety of input features as it is our case of genomics where different clusters of data might be identified; for instance as mutation (mu), methylation (met), copy number (cn) and/or drugs’ compounds (dc); the application of a covariance kernel parameterised with a unique length-scale results restrictive to the model. Restrictive in the following sense: let us suppose that the features among the mutation group smoothly covariate then a large length-scale could appropriately account for their covariance behaviours, whilst the features among methylation aggressively covariate then a short length-scale might adequately explain such a behaviour. Thus, if we chose to fit the model with a unique hyper-parameter length-scale this might not be able to sufficiently account for the contrastive behaviours among mu and met features. Hence, a particular kernel per group of features allows the model to have more flexibility by introducing a length-scale hyper-parameter to account for each type of feature. Therefore, instead of using a unique length-scale along all the features we build our kernel through a product of EQ kernels of the form^55^,

7 $$\begin{array}{l}k(x,x^{\prime} )={k}_{{mu}}(x,x^{\prime} )* {k}_{{met}}(x,x^{\prime} )* {k}_{{cn}}(x,x^{\prime} )* {k}_{{dc}}(x,x^{\prime} ),\\ k(x,x^{\prime} )=\exp \left(-\frac{{{|}}{x}_{{mu}}-x^{\prime}_{{mu}}{{{|}}}^{2}}{{{2l}^{2}}_{{mu}}}\right)* \exp \left(-\frac{{{|}}{x}_{{met}}-x^{\prime}_{{met}}{{{|}}}^{2}}{{{2l}^{2}}_{{met}}}\right)* \exp \left(-\frac{{{|}}{x}_{{cn}}-x^{\prime}_{{cn}}{{{|}}}^{2}}{{{2l}^{2}}_{{cn}}}\right)\\ \qquad \qquad \,\,* \exp \left(-\frac{{{|}}{x}_{{dc}}-x^{\prime}_{{dc}}{{{|}}}^{2}}{{{2l}^{2}}_{{dc}}}\right),\,\end{array}$$

where each kernel has a sub-index label to indicate its particular operation over the features related to the label, for instance ${k}_{{mu}}(x,x^{\prime} )$ only operates over the mutation features, ${k}_{{met}}(x,x^{\prime} )$ only operates over the methylation features and so on. The application of a particular kernel per type of feature allows the model to have more flexibility by introducing a length-scale hyper-parameter to account for each type of feature, instead of using a unique length-scale along all the features.

For the first experiment we built a model per each drug using cross validation with Kfolds = 20, the rank *R* was cross-validated from $\{1,...,D\}$, the coefficients ${a}_{d,i}$ were sampled from a normal distribution $N(\mathrm{0,1})$, each length-scale hyper-parameter was sampled from a uniform distribution with range $(\mathrm{0.01,3}\sqrt{24}),$ we used a total of 840 seeds for sampling the coefficients ${a}_{d,i}$ and length-scales. It is important to highlight that for the kernel product in Eq. (7), we used only two kernels, ${k}_{{mu}}(x,x^{\prime} )$ for mutation features (11 features) and ${k}_{{cn}}(x,x^{\prime} )$ for copy number features (13 features); this is due to the benchmark ANOVA method only having been applied to those types of features.

For the second experiment we built a model for each cancer type, we split the data in 70% training and 30% testing dose curves, ensuring that the amount of drugs present in the complete dataset is proportional to the amount in the training and testing sets. From the training data we performed a process of increasing the number of dose–response curves used for fitting the MOGP model. The increment of the dose–response curves for fitting the MOGP is as follows: $\{\mathrm{8,16,27,42,58,74}\}$ for Breast, $\{\mathrm{7,15,26,42,57,72}\}$ for COREAD, $\{\mathrm{10,19,34,53,72,91}\}$ for LUAD, $\{\mathrm{10,20,35,54,74,93}\}$ for melanoma and $\{\mathrm{9,18,32,51,69,87}\}$ for SCLC; the dose–response curves are randomly selected from the complete training set, we run six different random seeds to sample each value of increment. To find the best model for each increment (at each random seed) we cross validated with Kfolds = 5, the rank $R$ was cross-validated from $\{1,...,D\}$, the coefficients ${a}_{d,i}$ were sampled from a normal distribution $N(\mathrm{0,1.5}),$ each length-scale hyper-parameter was sampled from a uniform distribution with range $(\mathrm{0.05,1.5}\sqrt{780}),$ we used a total of 108 seeds for sampling the coefficients ${a}_{d,i}$ and length-scales. Unlike the type-1 experiment, for this type-2 experiment we have all the categories of features, mutation $({mu})$, methylation $({met})$, copy number $({cn})$ and drugs’ compounds $({dc})$; thus we apply the kernel product presented in Eq. (7), where ${k}_{{mu}}(x,x^{\prime} )$ operates over 279 features, ${k}_{{met}}(x,x^{\prime} )$ over 418 features, ${k}_{{cn}}(x,x^{\prime} )$ over 71 features, and ${k}_{{dc}}(x,x^{\prime} )$ over 12 features (refer to the “Range selection for the uniform distributions to initialise the length-scale hyper-parameters” section for detailed information on the initialisation of the length-scale hyper-parameters”).

For both types of experiments we optimised the kernel hyper-parameters, the coefficients ${a}_{d,i}$ and the likelihood noise parameter ${\sigma }^{2}$ by means of the LBFGS optimiser^56^. All the training of the MOGP models was performed using the python library: GPy (version 1.0.7) and Numpy (version 1.21.2).

### Range selection for the uniform distributions to initialise the length-scale hyper-parameters

The choice of ranges for the uniform distributions of the length-scale hyper-parameter is based on the fact that we expect the length-scale to be positive and up to a certain value that covers possible covariances between the input space features (genomics and drug compounds features in our case). For instance, the choice of $(\mathrm{0.05,1.5}\sqrt{780}),$ ranges from a small length scale $0.05$ up to $1.5\sqrt{780},$ where the upper limit is selected as a rule of thumb when dealing with high dimensionalities, in this case the dimensionality is $P=780$. This rule of thumb provides a sensible way to initialise a length-scale that fits the spread of the input features across all dimensions. With this approach we assume that a length-scale proportional to $\sqrt{P}=\sqrt{780}$ is feasible for capturing covariances in a high dimensional space. On the other hand, the choice of number of seeds is just the number of possible models that we run in a High Performance Computing (HPC) server to cross-validate and select the best model. The number of seeds used to cross-validate was a trade-off between the time complexity of the model and HPC resources available. The more HPC resources one can access the more alternative initialisation models one can cross-validate. Nonetheless, we found that among the different models cross-validated we were able to find various of them with similar and salient performances.

### Prediction on GDSC1 and GDSC2 and vice versa

In the interest of analysing the capabilities of our model, we explore two scenarios of the type-1 dataset: for the first scenario, we assume the GDSC1 dataset as the training source and the GDSC2 as the testing one; for the second scenario we assume the contrary, GDSC2 is assigned as the training source and GDSC1 as the testing. It is worth mentioning that the drug concentration doses are not the same for GDSC1 and GDSC2, thus we have to measure the prediction just at the region where both curves overlap. Supplementary Figure 16 shows an example of the different concentrations in the drug response curves for GDSC1 and GDSC2 datasets. The MOGP models would predict the cell viability per drug concentration, i.e., for GDSC1 the MOGP would predict $D=9$ outputs, and for GDSC2, $D=7$ outputs. We interpolate the outputs to render the full curves in order to extract the summary metrics AUC, IC50 and Emax.

### Estimating AUC, IC50 and E-max for observed and predicted dose–responses

To compute the Area Under the Curve (AUC), the drug concentration to achieve 50% cell viability (IC50), and the fraction of viable cells at the highest drug concentration (Emax), is necessary to evaluate a function $f(c)$ that describes the dose–response curve amongst the range of minimum drug concentration and maximum drug concentration, i.e., $c=[{MinDC},{MaxDC}]$. One can compute the AUC through the trapezoidal rule,

8 $${AUC}={\int_{a}^{b}}f(c){dc}\,\approx \,\mathop{\sum}\limits_{i=1}^{I}\frac{f({c}_{i-1})+f({c}_{i})}{2}\bigtriangleup {c}_{i},$$

where each ${c}_{i}$ represent the $i$-th value of a grid that partitions the range $[a,b]$ into $I+1$ points with partition size, $\bigtriangleup {c}_{i}={c}_{i}-{c}_{i-1}$; and the range’s values are $a={MinDC}$ and $b={MaxDC}$. To compute the AUC presented in the equation above we used the function ‘auc’ from the python package: metrics, implemented in the library scikit-learn (version 1.0.2). On the other hand, we can compute the IC50 by just finding the drug concentration value ${c}_{{IC}50}$ for which $f({c}_{{IC}50})\,=0.5$, and the Emax is just the value of the function $f({c}_{{MaxDC}})$ evaluated at ${c}_{{MaxDC}}={MaxDC}.$

### Measuring observed dose–response curves

For each of the cell lines of our datasets we applied the sigmoid function with four parameters (Sig4) as the evaluating function $f(c)$ that describes our observed dose–response curves. This Sig4 function is our reference dose–response curve from which we extract the true labels AUC, IC50 and Emax for each cell line. The Sig4 can be expressed as:

9 $$f(c)=\frac{1.0}{L+\exp (-\tau * (c-{c}_{0}))}+d,$$

where $L,\,\tau ,\,{c}_{0}$ and $d$ are the four parameters that describe the curve, and $c$ is the drug concentration for which we expect to evaluate the range, $c=[{MinDC},{MaxDC}]$^57^.

### Measuring predicted dose–response curves

On the other hand, the MOGP model predicts the cell viability at $D$ concentrations as per the GDSC dataset, i.e., the MOGP generates $D$ predictions that can be expressed as $\hat{f}({c}_{1}),...,\hat{f}({c}_{D}),$ where ${c}_{d}$ represents the $d$-th drug concentration. In order to compute the summary metrics AUC, IC50 and Emax as previously described, one needs to interpolate such $D$ predictions and obtain a continuous $\hat{f}(c)$ function that describes our predicted dose–response curves. We applied the piecewise cubic hermite interpolating polynomial (PCHIP) method to obtain $\hat{f}(c)$. It is a shape preserving method that reduces oscillating behaviours in the interpolation. We used the function ‘pchip_interpolate’ from the python package: interpolate, implemented in the library scipy (version 1.0.2). From such an interpolated function $\hat{f}(c)$ we can then obtain the predicted AUC, IC50 and Emax metrics in the same way as for the observed dose–response curves.

It is worth mentioning that for our experiments we split the IC50 metric between non-responsive and responsive behaviours as follows: ${IC}50 > 1.0$ is non-responsive and ${IC}50\,\le \,1.0$ is responsive. Also, for the drug response curves, we used a normalised range of drug concentrations between $0.1428$ or $0.1111$ as the minimum drug concentration for $D=7$ or $D=9$ respectively and $1.0$ as the maximum drug concentration. Any value higher than the maximum is considered non-responsive and we label it as ${IC}50=1.5$ by default. When studying the melanoma cancer, a cancer very responsive to the drugs PLX-4720, SB590885 and Dabrafenib, we also split the AUC and Emax metrics between non-responsive and responsive behaviours as follows: ${AUC}\le 0.55$ is responsive and ${AUC} \,> \,0.55$ is non-responsive, and $E\max \le 0.5$ is responsive and $E\max > 0.5$ is non-responsive.

### Benchmark methods

#### SRMF

SRMF was used to benchmark our method. This method does not use train and test datasets, but instead takes as input a response matrix containing a mix of response values and NaN values. It then utilises the known values to predict a whole new drug response matrix. To work around this difference, for each pair of test and train datasets, we overrode a copy of the test matrix with NaN values and concatenated it with the train dataset. This was used as the input drug response matrix. This ensured that only values from the test datasets were seen, and thus used, by the model when making predictions, while still including the test data observations in the prediction. We then compared the test data drug response values to their corresponding predicted values from the SRMF output. The original SRMF model was designed for IC50, however, we also ran it for AUC and Emax values (we did not take the log of these beforehand). Pearson correlation was used to create the similarity matrix inputs for both cell lines and gene properties. As we used genetic features for our model rather than gene expression, to be consistent, we also used genetic features when calculating cell line similarity when running SRMF. The hyperparameter K was left at its default as it was originally chosen for GDSE data.

#### Lasso regression and elastic net

Lasso and Elastic Net have been implemented as the baseline methods. Lasso regression is the linear model with L1-norm while Elastic Net takes together the L1 and L2-norm as the penalty in loss function. We built the linear models under two different settings:

1. The model is trained and predicted separately on different drugs with one type of the genomics features;
2. The model is trained and predicted with multi-omics features and together with drug chemical features.

In setting 1, we aimed to investigate the contribution of different omics data in predicting the drug response^31^. In setting 2, we built the linear model in a multi-task learning fashion^8^, where multiple drugs are trained and predicted in one model by taking the drug chemical features. We provided the performance metrics for both the single-task-single-omics and multi-task-multi-omics models.

#### DeepCDR

DeepCDR is a deep learning (DL) method integrating gene expression, mutation, and DNA methylation profiles. In our investigation, we opted for mutations, copy number alterations, and DNA methylation profiles as our cell line input features. To ensure a consistent comparison, we replaced gene expression with copy number alteration. For the drug encoders, we applied the uniform-graph convolutional neural networks as is designed in the original paper. Throughout the training phase, we implemented a 3-fold cross-validation strategy with early stopping. The performance evaluation was conducted on a test set that was distinct from the training and validation sets yet identical to the test sets used in benchmarking with other methods.

#### GraphDRP

GraphDRP is a DL method taking mutational profiles and drug chemical graph as input to predict the drug response. In the original implementation, three GNNs are compared as the drug encoder: GCN, GAT and GIN, while GIN was reported to have the best performance. Consequently, GIN was selected for encoding drug features in our analysis. The same cross-validation and early stop as DeepCDR is applied during the training phase. Performance evaluation was systematically conducted on the same test sets, ensuring comparability of results.

#### NeRD

NeRD is a DL model that uses miRNA and copy number as cell line features, while both chemical graph and drug fingerprints are used for drugs. In our evaluation, mutation profiles substituted miRNA data to align with our benchmark. The same cross-validation and early stop as DeepCDR is applied during the training phase. The performance is evaluated on the testing set.

## Supplementary information

Supplementary Information

Supplementary Data 1

Supplementary Data 2

Supplementary Data 3

Supplementary Data 4

## Data Availability

The drug response data is downloaded from the Genomics of Drug Sensitivity in Cancer (http://www.cancerrxgene.org). Drug chemistry data is downloaded from the pubchem website (https://pubchem.ncbi.nlm.nih.gov/). 24 melanoma-specific CFEs (genetic variations and CNAs) detected in the associated cell lines were obtained from https://ftp.sanger.ac.uk/pub/project/cancerrxgene/releases/release-8.4/GDSCtools_mobems.zip. The ANOVA statistical analysis to determine associations between cancer features and drug sensitivity, reported in the GDSC database is downloaded from (https://cog.sanger.ac.uk/cancerrxgene/GDSC_release8.4/ANOVA_results_GDSC1_24Jul22.xlsx and https://cog.sanger.ac.uk/cancerrxgene/GDSC_release8.4/ANOVA_results_GDSC2_24Jul22.xlsx). Genomic annotation data of a panel of 1001 human cancer cell lines were obtained from the GDSC1000 resource site https://www.cancerrxgene.org/gdsc1000/GDSC1000_WebResources///Data/BEMs/CellLines/CellLines_Mo_BEMs.zip. The paper and the Supplementary Information contain all necessary data to assess the conclusions. Supplementary data are provided with this paper.

## Code availability

All source code to preprocess the data, fit the models and generate the results shown in this paper is publicly available at https://github.com/juanjogg1987/MOGPs_for_Dose_Response_Predictions.
