# Supplementary material for: Multi-output prediction of dose–response curves enables drug repositioning and biomarker discovery
Source: NPJ Precis Oncol. 2024 Sep 20;8:209. doi: 10.1038/s41698-024-00691-x (PMC11415488; doi:10.1038/s41698-024-00691-x)
Supplement: Supplementary file 1 — Supplementary Information [file 41698_2024_691_MOESM1_ESM.pdf]

Supplementary Table 1. Comparison of existing dose-response prediction methods to the proposed multiple output Gaussian Process (MOGP).

Method	Feature selection	Predicts	Datasets	Language	Source code	Input features	Learning approach
FRF[1]	Intrinsic - embedded in the random forest method	Whole curve	CCLE, GDSC1 (v5), HMS-LINCS	MATLAB	https://github.com/razrahman/Functional-Random-forest	Single-omic: gene expression for GDSC and CCLE, and protein expression from HMS-LINCS<br>Drug features: None	Inductive method for cell lines, and transductive methods for drugs
SRMF[2]	Chemical and genomic (gene expression) similarities between drugs and cell lines, respectively, calculated using spearman rank	IC50	CCLE, GDSC1	MATLAB	https://github.com/linwang1982/SRMF	Single-omcis: gene expression<br>Drug features: chemical fingerprint descriptors	Transductive method for drugs and cell lines
cwKBMF[3]	Genes were grouped by prior knowledge of pathways	IC50	CTRP, GDSC1 (v5)	MATLAB	https://github.com/Ammad-uddin/cwkbmf	Single-omcis: gene expression, and prior knowledge of genetic pathways from MSigDB	Inductive method for both drugs and cell lines
QSAR-KBMF[4]	Kernel-based nonlinear dimension reduction	IC50	GDSC1	Matlab	https://research.cs.aalto.fi/pml/software/kbmf/	Multi-omics: gene expression, copy number and mutation<br>Drug features: chemical and structural descriptors	Inductive method for both drugs and cell lines
DeepCDR[5]	Convolutional neural networks and graph neural network.	IC50	GDSC1	Python	https://github.com/kimmo1019/DeepCDR	Multi-omics: Gene expression, mutation, and DNA methylation.<br>Drug features: drug chemical graph	Inductive method for both drugs and cell lines
GraphDRP[6]	Convolutional neural networks and graph neural network.	IC50	GDSC1	Python	https://github.com/hauldhut/GraphDRP	Single-omics: Mutational profile.<br>Drug features: drug chemical graph	Inductive method for both drugs and cell lines
NeRD[7]	Deep autoencoder, convolutional neural networks and graph neural network.	IC50	PRISM	Python	https://github.com/Shaw66/NeRD	Multi-omics: miRNA and copy number.<br>Drug features: drug chemical graph and fingerprints..	Inductive method for both drugs and cell lines

Supplementary Table 2. List of molecular descriptors, illustrating all the chemical features used along with their definitions, which are sourced from PubChem

Molecular Descriptor	Definition
2bonds	Number of double bonds
h_bond_acceptor_count	Number of hydrogen bond acceptors
rotatable_bond_count	Number of Hydrogen bond donors
molecular_weight	Molecular weight
complexity	Rating of complexity of structure, calculated using the Bertz/Hendrickson/Ihlenfeldt formula
surface_area	Polar surface area estimate
xlogp	Measure of hydrophobicity or hydrophilicity
bond_stereo_count	Number of bond stereocenters ?
heavy_atom_count	Number of heavy atoms (non-hydrogen atoms)
F, Cl, S	Presence or absence of specified elements in compound

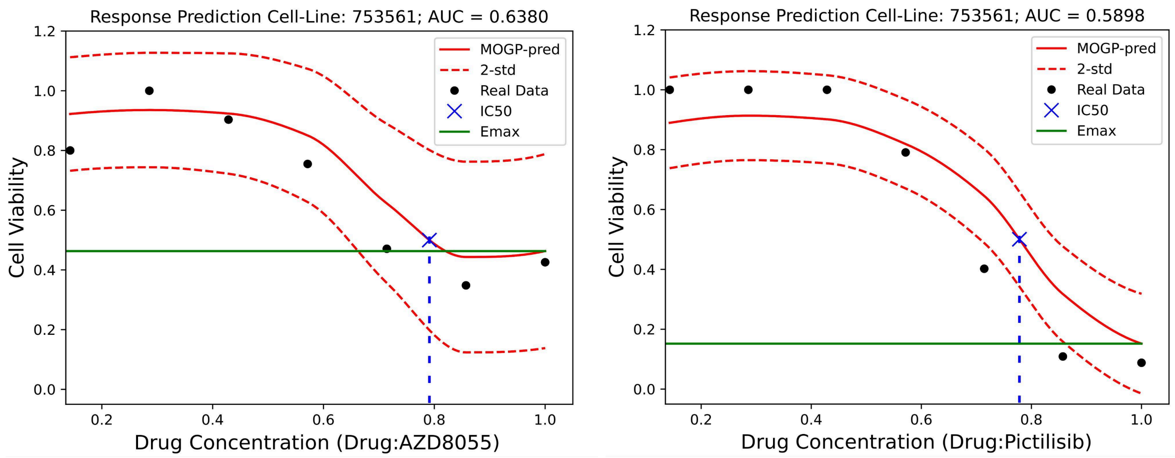

Supplementary Figure 1. Comparison of predicted whole dose response behaviours across drug concentrations in treating the same cell-line (COSMIC ID: 753561) with different drugs AZD8055 (left) and Pictilisib (right). By predicting the whole curve, we are able to see that these two cases have similar AUC and normalised IC50 values but different Emax values.

Biomarkers are robust between drug screening studies

It is important to highlight that the KL-Relevance determination method is meant to be computed over the same data used to train the MOGP model, although, here we are exploring what is the relevance of features to predictions on a separate screening study. Here, we use the MOGP trained on GDSC2 to obtain a KL-Relevance on the testing GDSC1 dataset. Also, we apply the reverse test, i.e., use the MOGP trained on GDSC1 to obtain a KL-Relevance on the testing GDSC2 dataset, which contains additional cell lines. Supplementary Figure 2 shows a comparison of the feature ranking obtained by the KL-relevance method when training the MOGP model with GDSC1, but computing the KL-relevance over GDSC2, and vice versa. The figure presents the features organised with respect to the average KL-relevance between GDSC1 and GDSC2; from left to right we sort according to high and low averages.

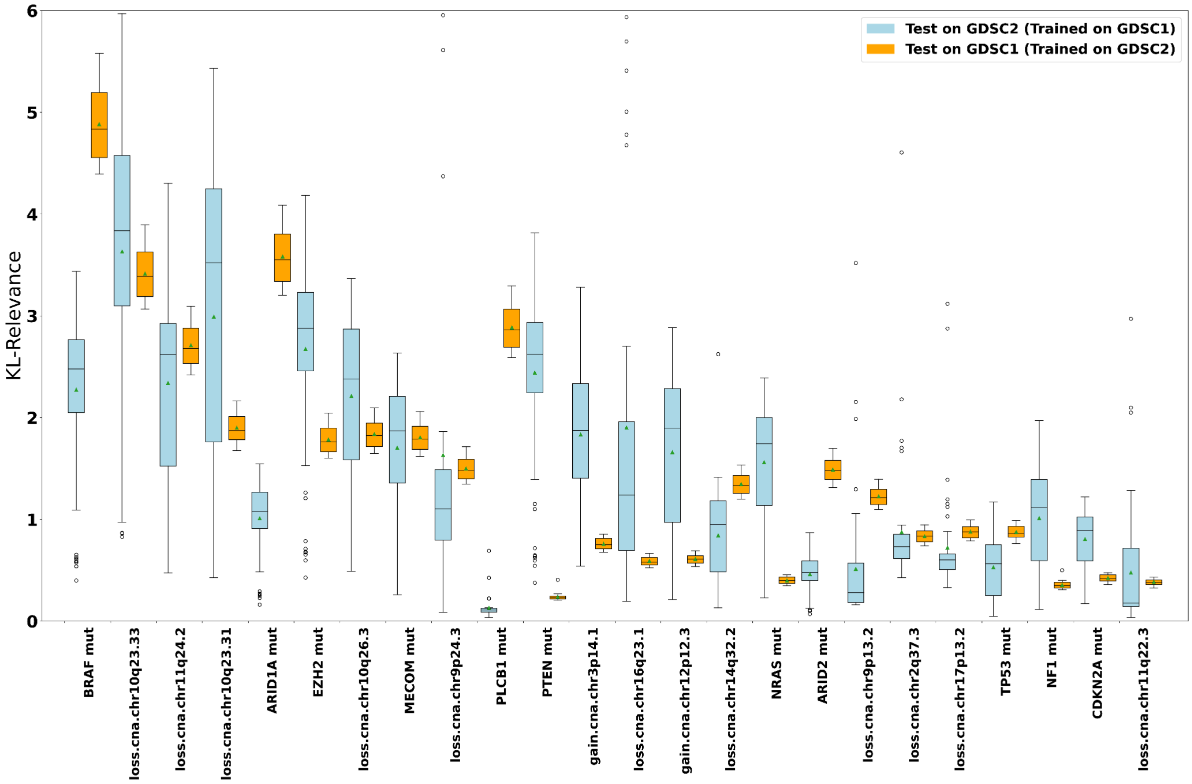

Supplementary Figure 2. Comparison of the feature ranking obtained by KL-relevance method when training the MOGP model with GDSC1, but computing the KL-relevance over GDSC2, and vice versa for drug PLX-4720. Each box represents the KL-relevance distribution of each feature w.r.t all the data in either GDSC1 (orange) or GDSC2 (light blue). From left to right the features are organised from high to low averages between the distributions' means (triangles in green colour) of GDSC2 and GDSC1 per feature.

The boxplots represent the KL-Relevance distribution per feature with respect to all the cell lines tested. The wider the boxplot the broader the KL-Relevance values obtained along the cell lines; this could imply that the model considers that for some cell lines such a feature is quite important, but for other cell lines the same feature is not that important for predicting the dose response curve. Generally the model trained on GDSC2 (tested on GDSC1) shows

to be more certain about the feature relevance by achieving narrower boxplot distributions in comparison to the model trained on GDSC1 (tested on GDSC2). There are some notable discrepancies between the mean KL-Relevance (green triangles) for some features. For instance, respectively for GDSC1 and GDSC2 models the BRAF mutation feature attains mean KL-Relevance metrics of roughly 2.2 and 4.8, loss.cna.chr10q23.31 of 1.8 and 3.0, ARID1A of 1.0 and 3.6, PLCB1 of 0.1 and 2.8, and PTEN of 2.5 and 0.2. In contrast, the features with smaller discrepancies are loss.cna.chr10q23.33, MECOM, loss.cna.chr9p24.3, loss.cna.chr2q37.3, loss.cna.chr17p13.2, TP53 and loss.cna.chr11q22.3 with an absolute difference of less than 0.3.

We believe that the model for GDSC2 reaches narrower boxplots with a more certain relevance determination due to having a larger number of cell lines available for training in comparison to the model trained on the GDSC1 dataset; also the GDSC2 dataset covers a broader range of drug concentration, $[0.01\mu M, 10\mu M]$, than the GDSC1 dataset with $[0.057\mu M, 10\mu M]$ as shown in the Supplementary Figure 11. Therefore, the MOGP model trained on the GDSC2 dataset (tested on GDSC1) is a more trustable predictor of the features ranking as well as the dose response curves.

Ranking comparison between KL-Relevance, ANOVA and SHAP methods

In order to broaden our comparative results we also provide a features ranking by applying the Shapley additive explanations approach (SHAP) [8]. The SHAP method receives the input features x (the genomic features and drug compounds in our case) together with the output's point estimate (the dose response in our case) predicted by a machine learning model. Nonetheless, our MOGP model does not particularly provide a point estimate prediction of the output; the MOGP predictions are characterised by a mean prediction accompanied by a (co)variance metric that indicates how certain the model is about the prediction. For instance, a mean prediction with high (co)variance indicates high uncertainty, whilst a mean prediction with low (co)variance indicates that the prediction might be more trustable with low uncertainty. Therefore, to apply the SHAP method with our MOGP prediction we have to opt for neglecting the (co)variance prediction and just use the mean prediction as the point estimate. We make a comparison between the ANOVA, our KL-Relevance and SHAP methods for melanoma cancer from the GDSC2 dataset when using the drugs: Dabrafenib, PLX-4720 and SB590885. Supplementary Figure 3 shows the ranking comparison between the three methods.

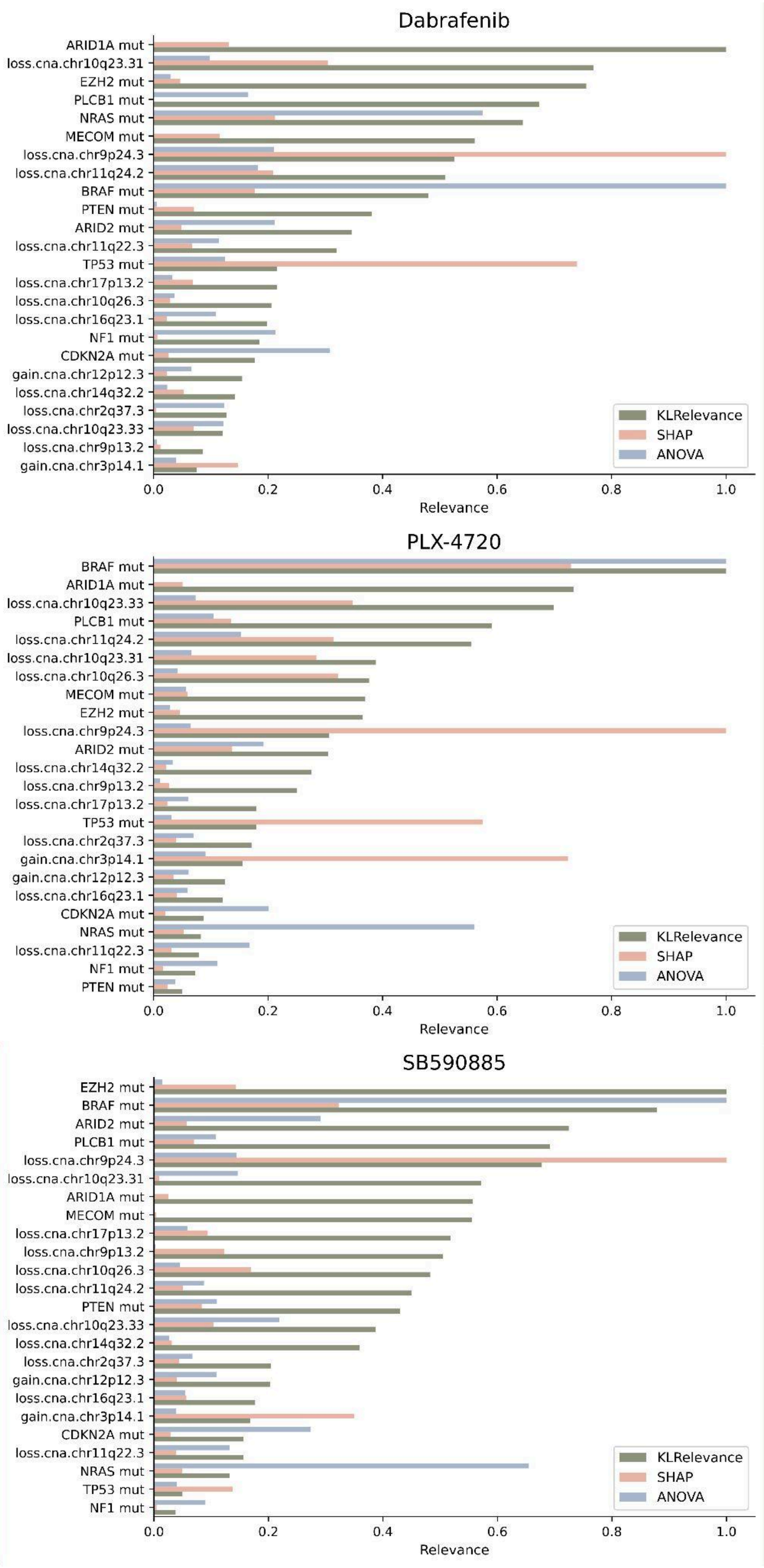

Supplementary Figure 3. Ranking comparison between KL-Relevance, ANOVA and SHAP methods for melanoma cancer from GDSC2 dataset when using the drugs: Dabrafenib, PLX-4720 and SB590885. The relevance values are normalised by dividing their magnitude values by their maximum; particularly ANOVA rankings are computed as -log10(ANOVA P-value), then normalised dividing by max(-log10(ANOVA P-value)).

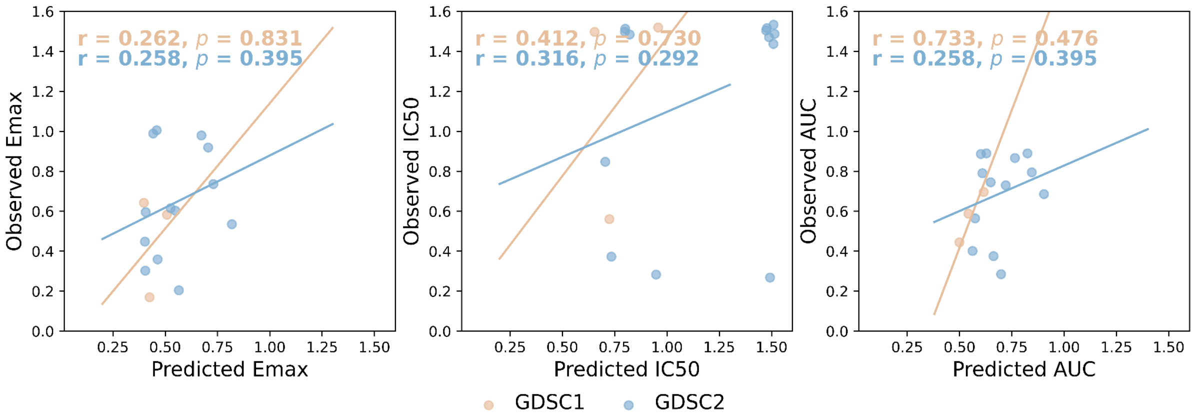

Supplementary Figure 4. Evaluation of the predictive performance of MOGP in predicting dose-responses of numerous melanoma cell lines treated with drug PLX-4720 only present in the GDSC1 (N=3) or GDSC2 (N=13). Three drug response metrics: Emax, IC50, and AUC were obtained from predicted dose-responses and compared with values derived from observed curves. The predictive power of the MOGP model was measured using Pearson correlation coefficient, *r*, to compare the values of MOGP-predicted and observed dose-response metrics.

Dose-specific prediction of drug response

Since samples from certain cancer types are difficult to acquire and drug screening experiments are expensive, in the second experiment we examined how the MOGP model's prediction performance varied with different numbers of dose response curves (DRCs) for training and reported the performance over the test set. We want to recall that the gradual increment of the dose response curves for fitting the MOGP model is as follows: $\{8, 16, 27, 42, 58, 74\}$ for BRCA, $\{7, 15, 26, 42, 57, 72\}$ for COREAD, $\{10, 19, 34, 53, 72, 91\}$ for LUAD, $\{10, 20, 35, 54, 74, 93\}$ for SKCM and $\{9, 18, 32, 51, 69, 87\}$ for SCLC. We used six different random seeds to sample each training set that was gradually incrementing in size. It is worth mentioning that the cancers were chosen as per their responsiveness to the drugs PLX-4720, SB590885 and Dabrafenib. Melanoma is very responsive (approx. 55% of the data), followed by COREAD being seldom responsive (approx. 9% of the data) and breast being hardly ever responsive (less than 3% of the data) and; LUAD and SCLC as non-responsive cancers at all.

Supplementary Figure 5 shows the prediction error of the MOGP model to forecast each of the seven dose concentrations whilst the number of DRCs in training is augmenting. From the figure we can see that the BRCA, COREAD and SKCM cancers generally present a monotonically decreasing trend in the error when increasing the number of DRCs in training. In contrast, the LUAD and SCLC cancers show an almost flatten behaviour when increasing

the curves used in training.

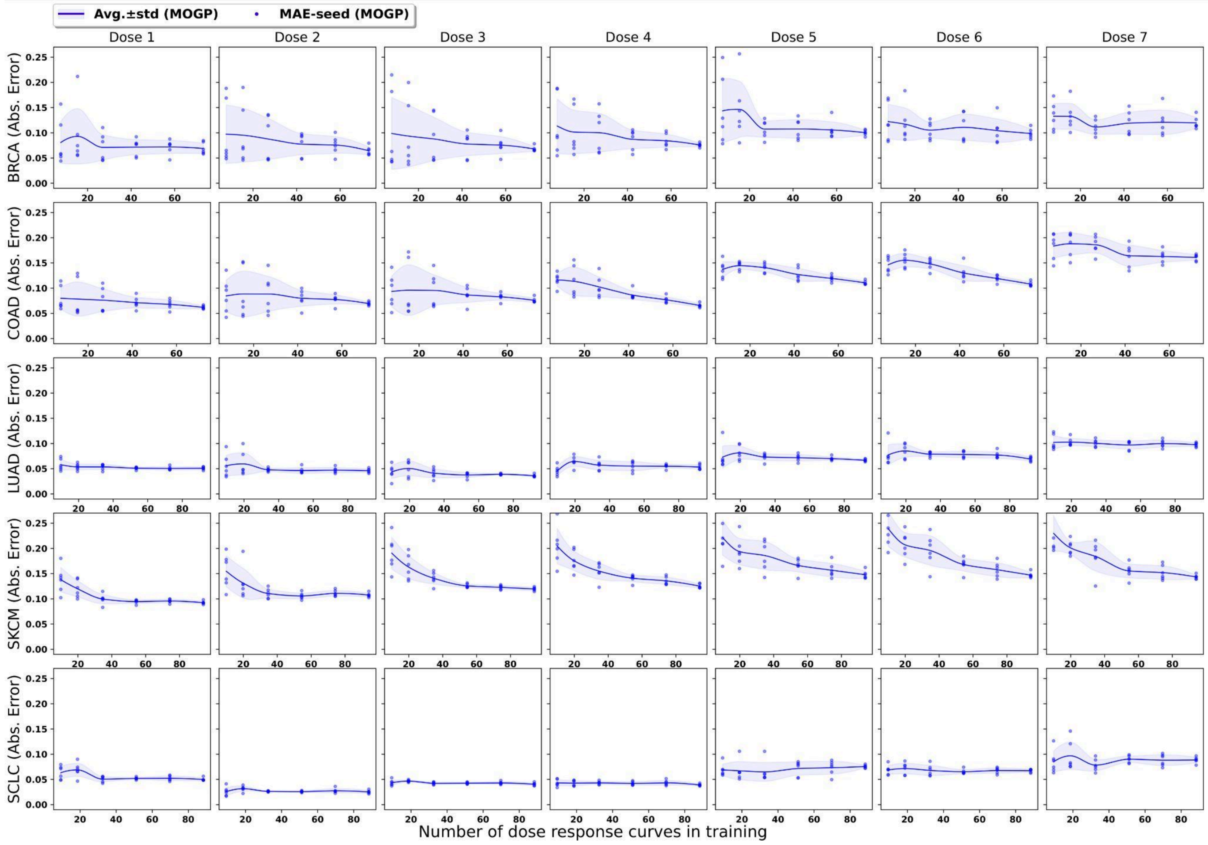

Supplementary Figure 5. Prediction error of the MOGP model when increasing the number of DRCs in training. From top to bottom: BRCA, COREAD, LUAD, SKCM and SCLC cancers. From left to right the subfigures present the prediction error of the dose concentration from dose 1 to dose 7. Each subfigure shows dots aligned vertically that correspond to the Mean Absolute Error (MAE) of the MOGP prediction per seed; i.e, the dots correspond to the different random seeds used to sample each training set that was gradually incrementing in size. Each gradual increment of curves in training involves six MAEs as per the six seeds, thus, along the number of DRCs in training, the blue line interpolates the averages of MAEs between the seeds at each increment.

Using a small number of DRCs in training there is a higher uncertainty in the model performance, especially in BRCA, COREAD and SKCM cancers. For instance, when the number of dose curves in training was less than 40 the Mean-error per seed appears more scattered in these cancers. Also, we can realise that doses 1, 2, 3 and 4 can be predicted achieving smaller errors than doses 5, 6 and 7. SKCM cancer is the most responsive cancer to the drugs used in the experiment, making it a more challenging cancer to model as shown in the prediction errors in comparison to the other cancers; the prediction error for SKCM is generally higher than the others.

Also it is important to highlight that, though generally the predicting performance of the model improves by increasing the curves in training, there are some doses that plateau at a particular point and some others that keep improving. For example, for BRCA cancer, doses 2, 3 and 4 keep improving their performance, but doses 1, 5, 6 and 7 roughly plateau after 40 DRCs in training. For COREAD cancer the prediction error for all doses keeps improving

when increasing the curves, but except the dose 7 that plateaus at around 60 dose responses in training. For SKCM cancer doses 1 and 2 try to plateau roughly after 50 curves in training, but all the remaining doses keep improving their prediction error when increasing the number of curves in training. We believe that the behaviour in BRCA cancer with many doses quickly flattening their predictive error is similar to the non-responsive cancers LUAD and SCLC due to the dataset having less than 3% of responsive curves to the drugs.

Error prediction of summary metrics for SKCM cancer: splitting dose curves by responsive and non-responsive

The Supplementary Figure 6 shows that when increasing the number of curves in training both SRMF and MOGP models reduce their error for the non-responsive test data along all the AUC, Emax and IC50 metrics. Nonetheless, for the responsive test data only the MOGP model shows an improvement whilst the SRMF degrades its performance.

On the other hand, it is worth mentioning that the SRMF commonly struggled to reach metrics close to the BERK reference for the different cancers. Conversely, the MOGP model reached a salient performance with respect to the AUC metric, generally the average Mean-Error was quite close to the BERK metric (for BRCA and COREAD cancers) or even attained a lower error being under the mean and median BERK metrics (for LUAD, SCLC and SKCM cancers). Regarding the Emax and IC50 metrics the MOGP model obtained errors close to the BERK’s mean or between the BERK’s mean and median; for SKCM cancer there is still room for the model to improve performance in the responsive data scenario.

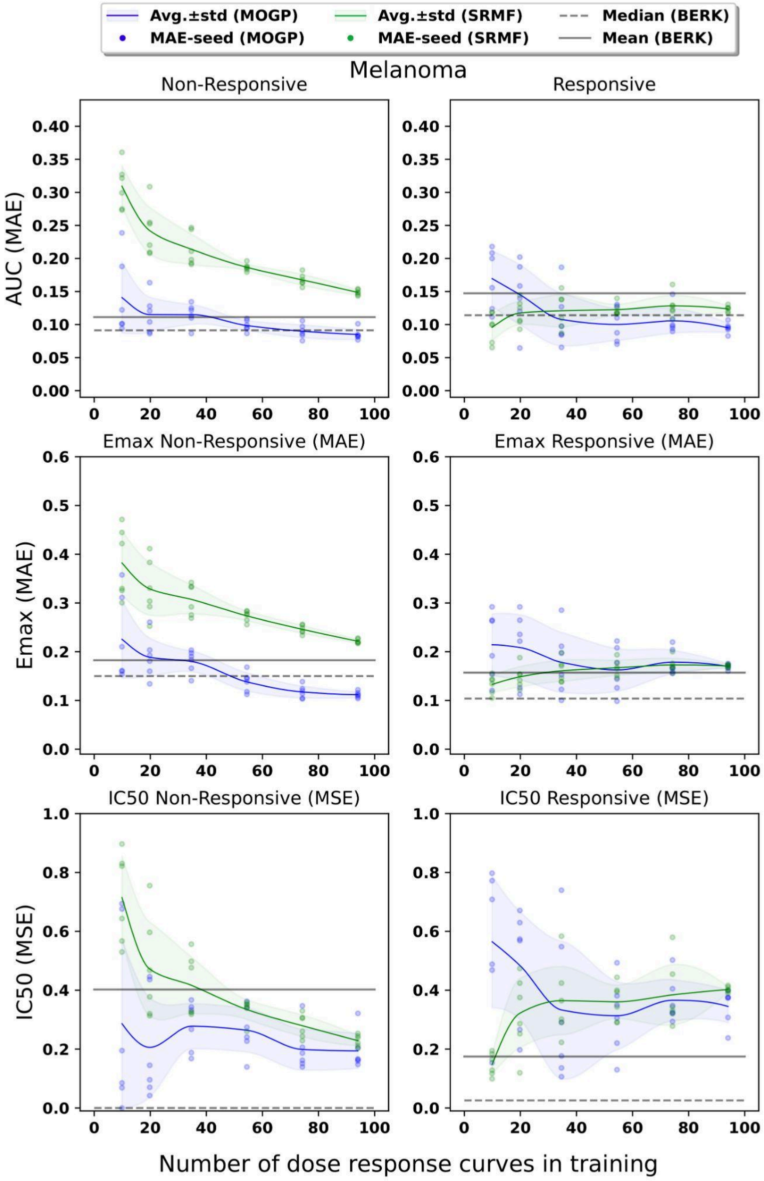

Supplementary Figure 6. Performance of Methods MOGP (blue colour) and SRMF (green colour) with respect to the summary metrics: AUC, Emax and IC50. The left and right columns depict the prediction errors over DRCs that are, as per the metric, responsive and non-responsive respectively. Each subfigure shows dots aligned vertically that correspond to the Mean Error of the methods per seed; i.e, the dots correspond to the different random seeds used to sample each training set that was gradually incrementing in size. Each gradual increment of curves in training involves six Mean Errors as per the six seeds, thus, along the number of DRCs in training, the solid line interpolates the averages of Mean Errors between the seeds at each increment. We split the metrics between non-responsive and responsive behaviours as follows: AUC>0.55 is non-responsive, Emax>0.5 is non-responsive, and IC50>1.0 is non-responsive. Thus, the figures titled non-responsive depict the errors for only the response curves in the test set that are non-responsive as per the metric, and the same for the figures titled as responsive.

Scaling up analysis to multiple cancer types and drugs targeting different biological pathwaysDrugs targeting different biological pathways

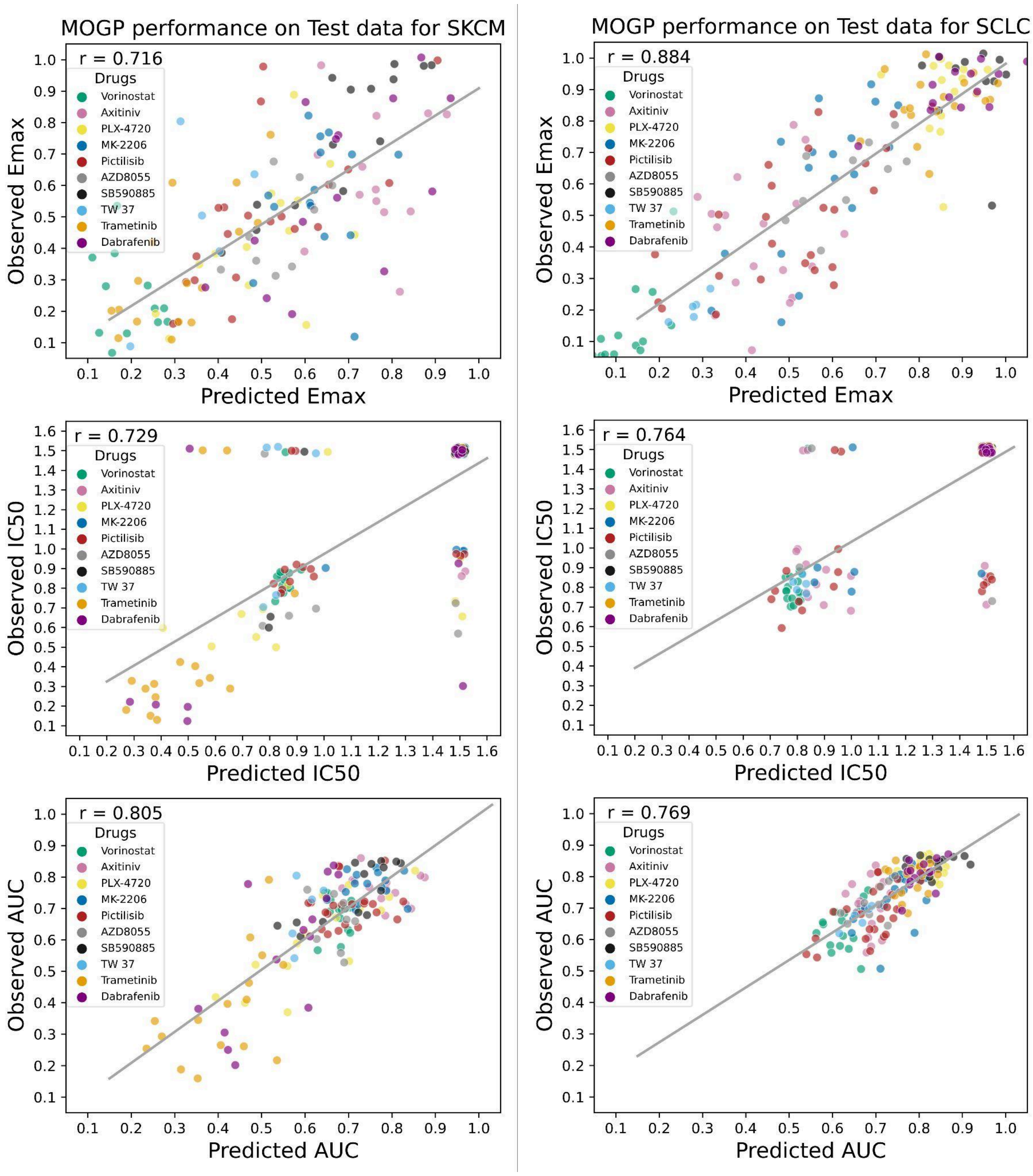

Supplementary Figure 7. Predictive performance of the MOGP model over testing data for SKCM and SCLC cancers. The Pearson correlation coefficient, r, is reported to compare three summary metrics Emax, IC50 and AUC derived from MOGP-predicted DRCs against observed dose-responses fitted using the sigmoid function with four-parameters. The experiments involved the prediction for 10 different drugs: Vorinostat, Axitinib, PLX-4720, MK-2206, Pictilisib, AZD8055, SB590885, TW 37, Trametinib and Dabrafenib.

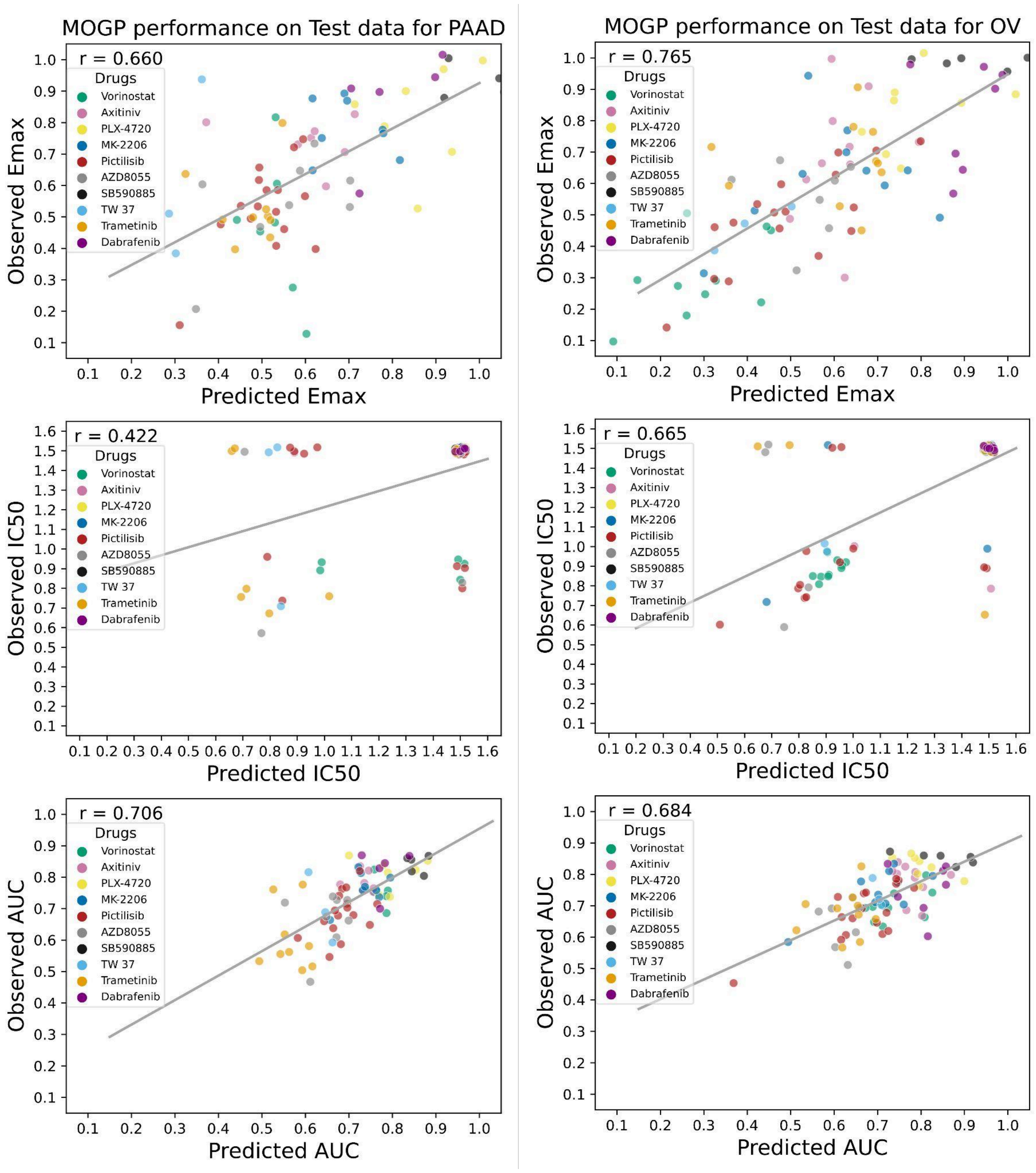

Supplementary Figure 8. Predictive performance of the MOGP model over testing data for PAAD and OV cancers. The Pearson correlation coefficient, r, is reported to compare three summary metrics Emax, IC50 and AUC derived from MOGP-predicted DRCs against observed dose-responses fitted using the sigmoid function with four-parameters. The experiments involved the prediction for 10 different drugs: Vorinostat, Axitinib, PLX-4720, MK-2206, Pictilisib, AZD8055, SB590885, TW 37, Trametinib and Dabrafenib.

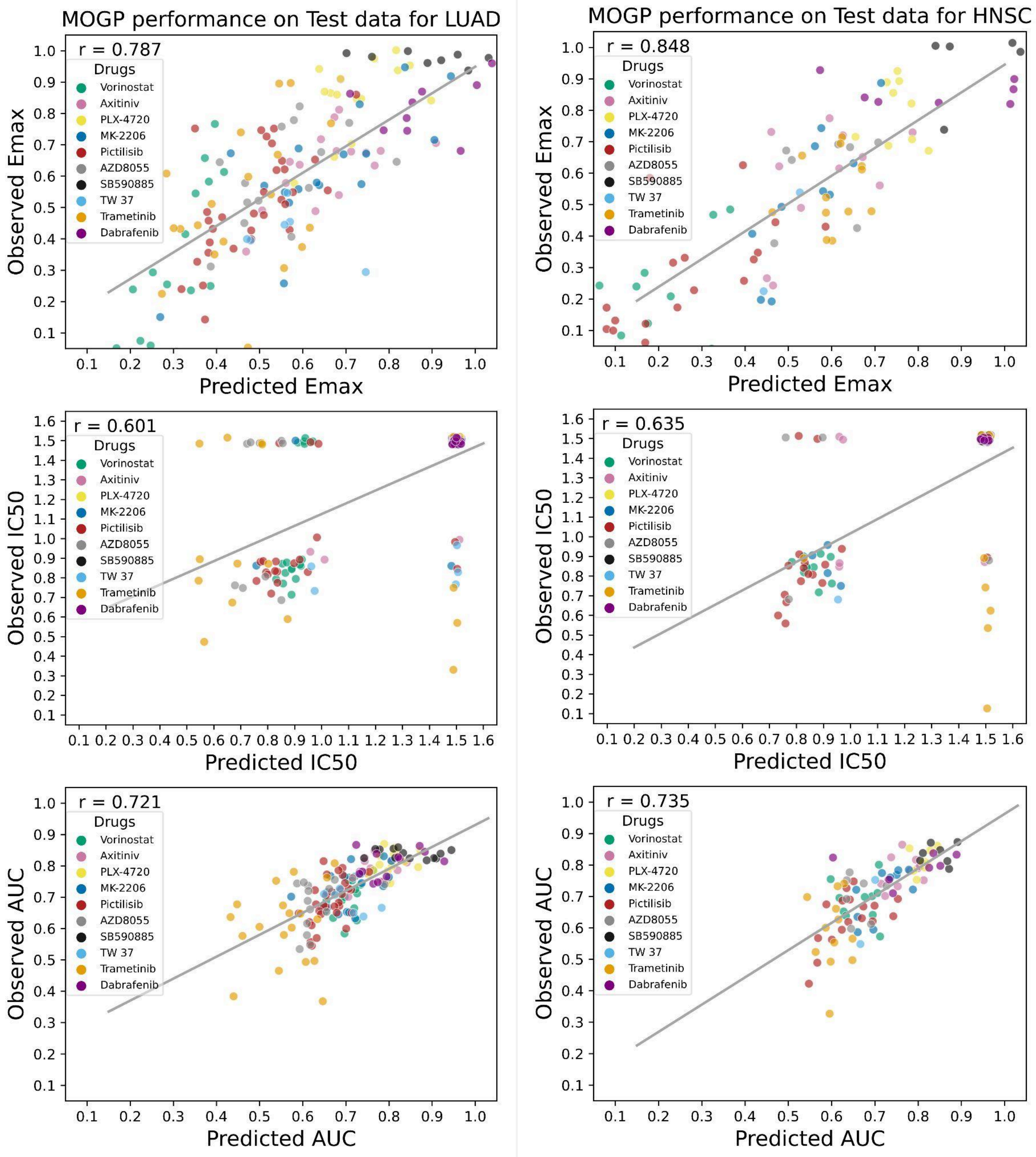

Supplementary Figure 9. Predictive performance of the MOGP model over testing data for LUAD and HNSC cancers. The Pearson correlation coefficient, r, is reported to compare three summary metrics Emax, IC50 and AUC derived from MOGP-predicted DRCs against observed dose-responses fitted using the sigmoid function with four-parameters. The experiments involved the prediction for 10 different drugs: Vorinostat, Axitinib, PLX-4720, MK-2206, Pictilisib, AZD8055, SB590885, TW 37, Trametinib and Dabrafenib.

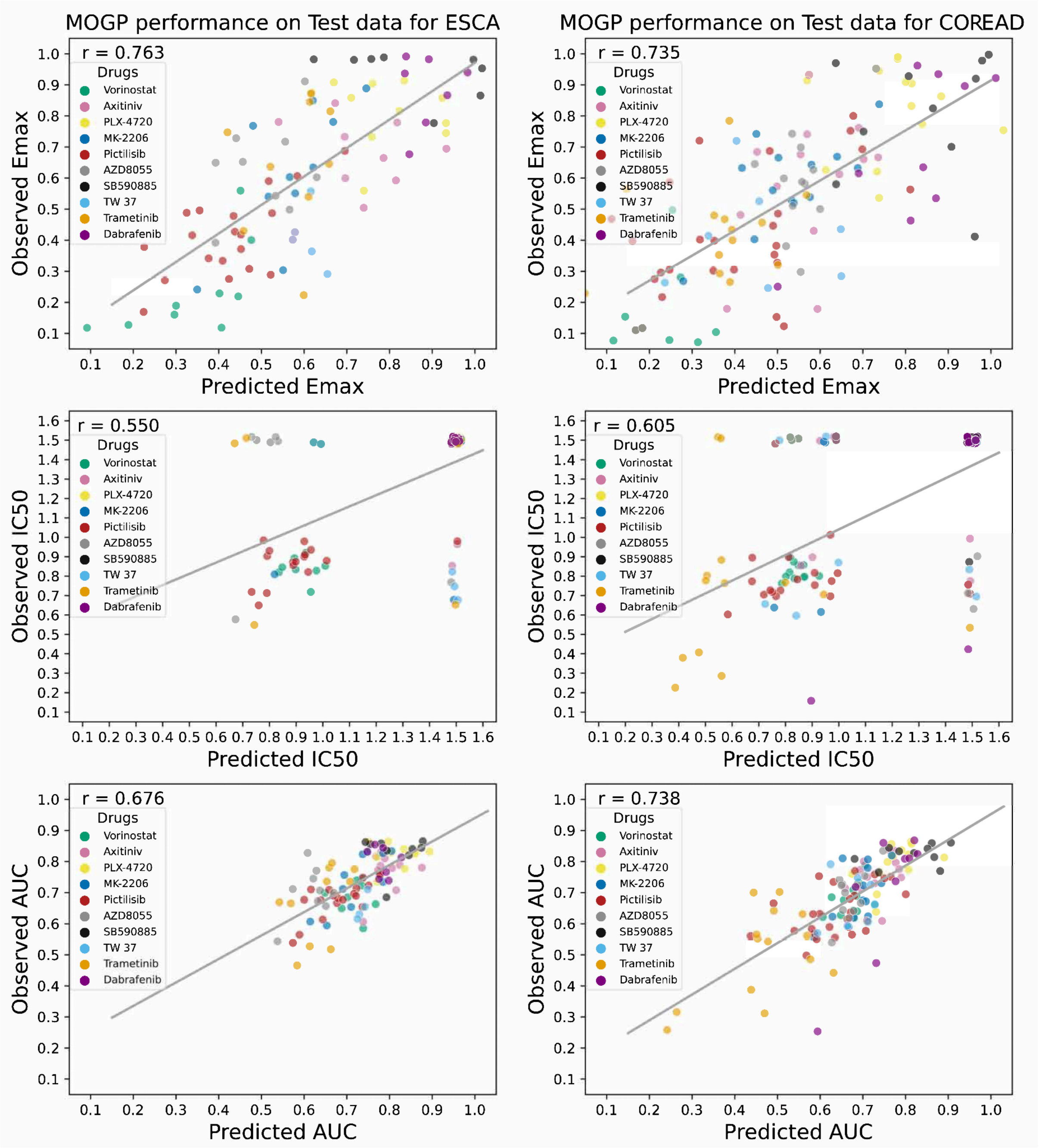

Supplementary Figure 10. Predictive performance of the MOGP model over testing data for ESCA and COREAD cancers. The Pearson correlation coefficient, r, is reported to compare three summary metrics Emax, IC50 and AUC derived from MOGP-predicted DRCs against observed dose-responses fitted using the sigmoid function with four-parameters. The experiments involved the prediction for 10 different drugs: Vorinostat, Axitinib, PLX-4720, MK-2206, Pictilisib, AZD8055, SB590885, TW 37, Trametinib and Dabrafenib.

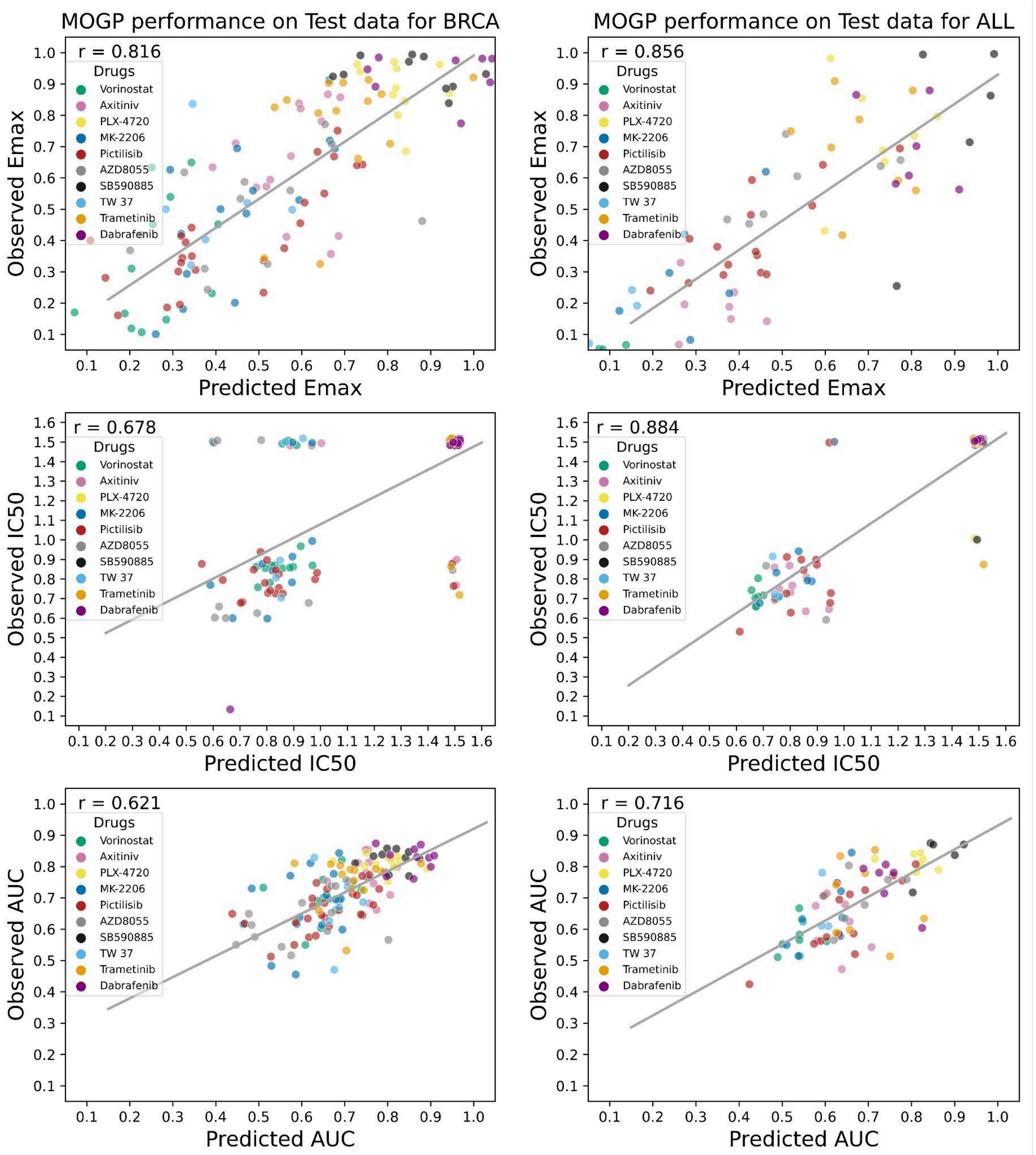

Supplementary Figure 11. Predictive performance of the MOGP model over testing data for BRCA and ALL cancers. The Pearson correlation coefficient, r, is reported to compare three summary metrics Emax, IC50 and AUC derived from MOGP-predicted DRCs against observed dose-responses fitted using the sigmoid function with four-parameters. The experiments involved the prediction for 10 different drugs: Vorinostat, Axitinib, PLX-4720, MK-2206, Pictilisib, AZD8055, SB590885, TW 37, Trametinib and Dabrafenib.

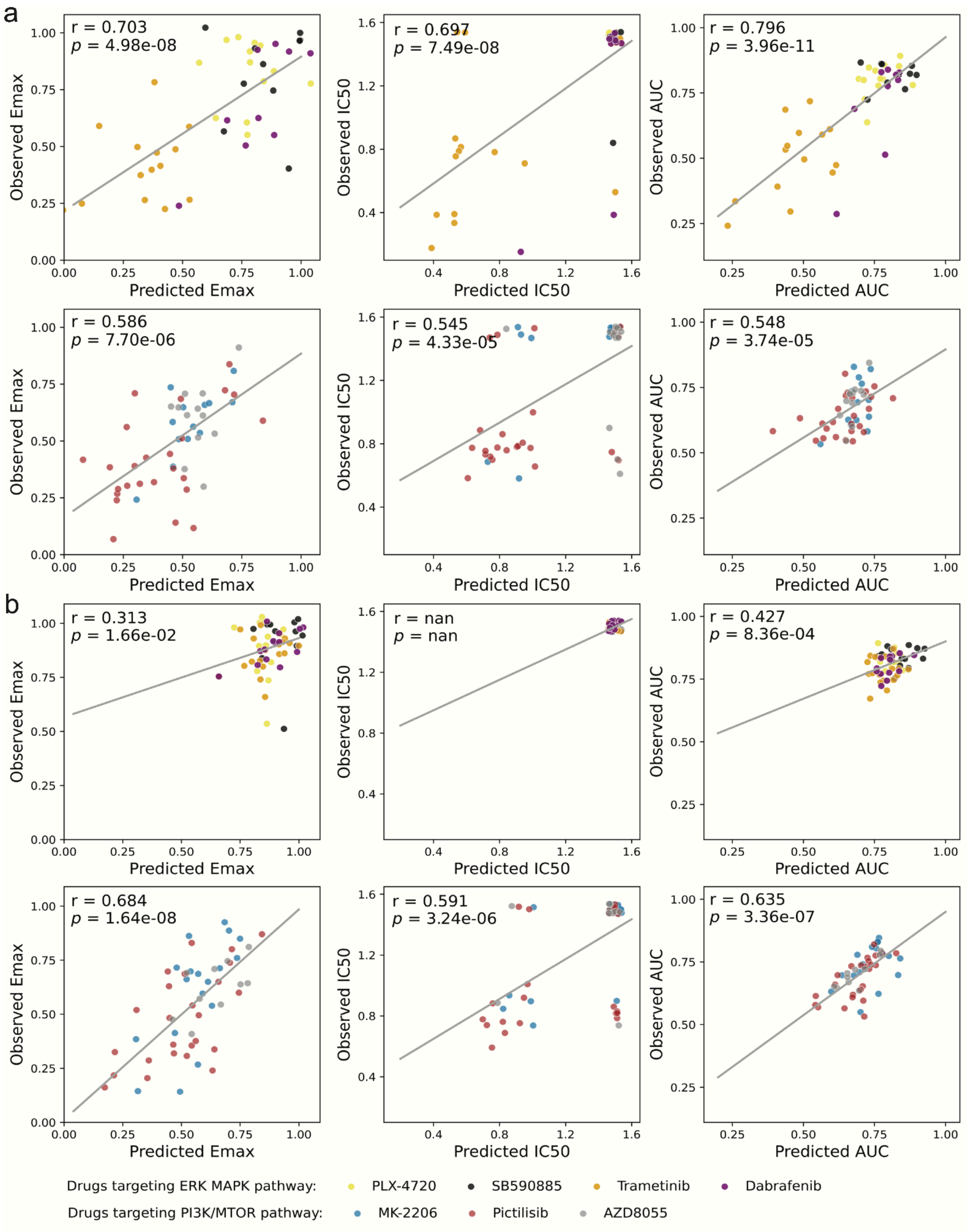

Supplementary Figure 12. The predictive power of MOGP model was assessed using Pearson correlation coefficient, r, comparing three summary metric values (Emax, IC50 and AUC) derived from MOGP-predicted DRCs against observed dose-responses fitted using the sigmoid function with four-parameters for both **a.** COREAD and **b.** SCLC cell lines treated with drugs targeting the ERK/MAPK signalling pathway (PLX-4720, SB590885, Dabrafenib and Trametinib), and PI3K/MTOR-targeted drugs (MK-2206, Pictilisib and AZD8805). COREAD cell line-drug pairs targeting the ERK/MAPK pathway demonstrated strong correlations between the predicted and observed values of Emax, IC50 and AUC. Robust correlations were also observed between predicted and observed metric values for

similar drugs targeting the PI3K/MTOR pathway in SCLC. However, correlation coefficients of these metrics only range between 0.30-0.43 in drugs targeting the ERK/MAPK signalling pathway in these SCLC cell lines.

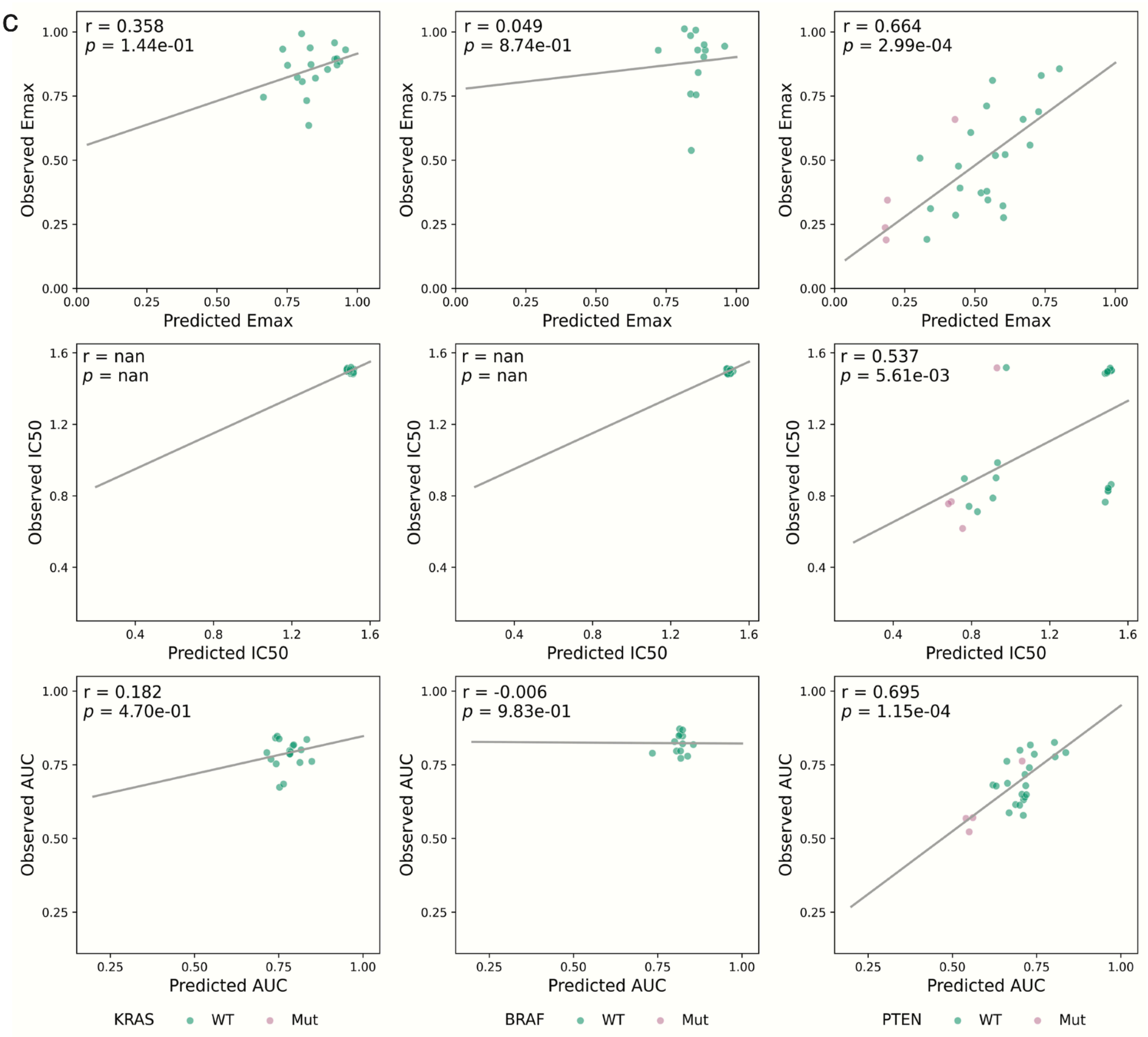

Supplementary Figure 13. The prediction of dose responses for the treatment of individual inhibitors of: i) MEK 1/2 (Trametinib), ii) BRAF (PLX-4720) and iii) PI3K-class 1 (Pictilisib) on SCLC cell lines was evaluated in relation to KRAS, BRAF or PTEN mutational status (wild-type: green; mutation: pink). Considering that *PTEN*, *TP53* and *RB1* mutations are more frequently found in SCLC [9], and the PI3K/MTOR signalling is among the most commonly affected pathway in this cancer [10,11], the predictive power of response curves for drugs targeting the ERK/MAPK pathway on these cell lines might be constrained by the limited availability of sensitive response data in the training dataset. These SCLC cell lines, harbouring wild-type status in KRAS and BRAF, are predominantly resistant to inhibitors targeting MEK 1/2 and BRAF within the ERK/MAPK pathway respectively, with lower correlation coefficients compared to Pictilisib, which targets the PI3K/Akt signal transduction.

Time performance to train the MOGP model on cancer datasets with different N sizes

To give an idea of the time consumption for training a MOGP model [12] for different cancer datasets with different sizes, we collect the times per model trained and provide a box plot of the time performance. We selected 10 cancer types and trained the models to predict the dose response for each cancer. By means of a cross-validation process we trained 44 models per each cancer type. We trained the models using an ADAM optimiser in a High Performance Computing system with 32 CPU cores and 24 GB of Ram memory. Supplementary Figure 14 shows the time distribution for training the MOGP model for each cancer type. As it can be seen from the figure below, once the number of dose responses increases then also the time consumption increases as per the model's computational complexity.

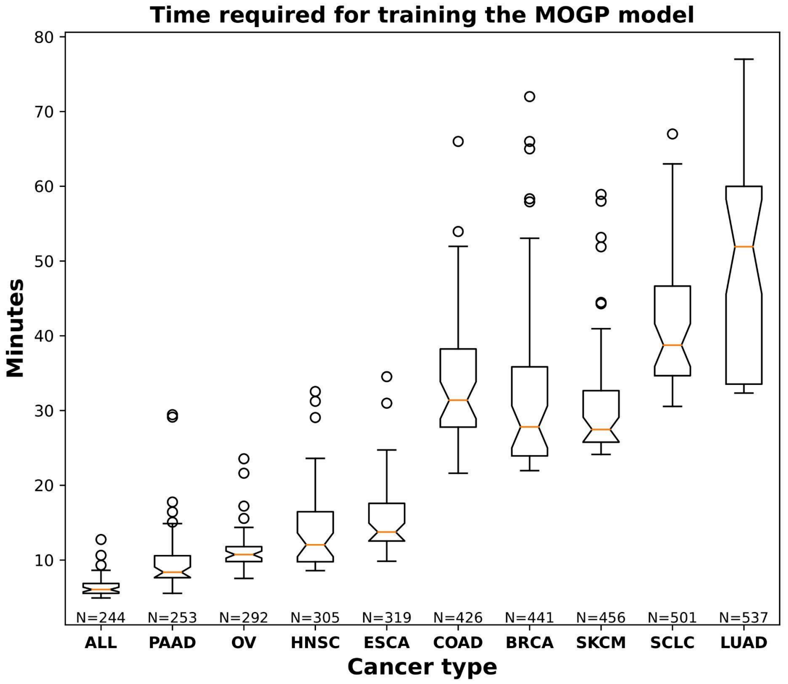

Supplementary Figure 14. Time performance to train the MOGP model for ten cancer types. Each box plot is generated with 44 models trained with different hyper-parameters initializations. The N values shown above the cancer names refer to the number of dose responses available for the cancer.

Train and test on different cancer types

In this experiment we aim to model the dose response of a cancer that presents very few data observations. Generally, for common cancers we might have access to many data observations, whilst in the context of rare cancers the lack of data is a usual situation. We want to explore if the data information known from common cancers can be transferred to improve predictions on a rare cancer, i.e. if the predictive performance of a model that targets the dose response of a cancer A (with very few observations) could be improved by means of feeding the model with dose responses from cancers different to such a cancer A. Thus, in the interest of emulating the rare cancer scenario and training our MOGP model

presented in the main manuscript, we selected Melanoma cancer as the cancer with very few observations and used other four cancers with many more observations. Our dataset consists of N dose response curves produced by the drugs PLX-4720, SB590885 and Dabrafenib: Melanoma ($N = 142$), Breast ($N = 110$), COREAD ($N = 108$), LUAD ($N = 139$), and SCLC ($N = 133$); all cancers present 780 features, from which we have 279 of mutation, 418 of methylation, 71 of copy number and 12 of drug compounds. Such $P = 780$ input features are meant to be mapped to predict seven drug concentrations ($D = 7$) that form the dose response.

We train our MOGP model to predict for Melanoma cancer, but our dataset is built with only 9 dose response curves from Melanoma plus dose responses from the other four remaining cancers. Since on average the cancers have an N = 126 dose responses, we are assuming to have, from the rare cancer, approximately 7% of dose response curves for training and 93% for testing. We do not use all the dose responses from the remaining four cancers, but gradually increment their number of dose response curves in training. The increment of the dose responses from the four remaining cancers is as follows: {0,48,96,192,384,576}; the dose responses are randomly selected and we run six different random seeds per value of increment.

From the MOGP prediction we extract the AUC, Emax and IC50 summary metrics. The Supplementary Figure 15 shows the predictive performance of the MOGP model as per the summary metrics for Melanoma cancer when increasing the number of dose response curves from the remaining four cancers. The box-plot per value of increment shows the error trend of the predictions; a blue line (with plus and minus a standard deviation) represents the average of the six mean-errors achieved by each of the models for the different random seeds.

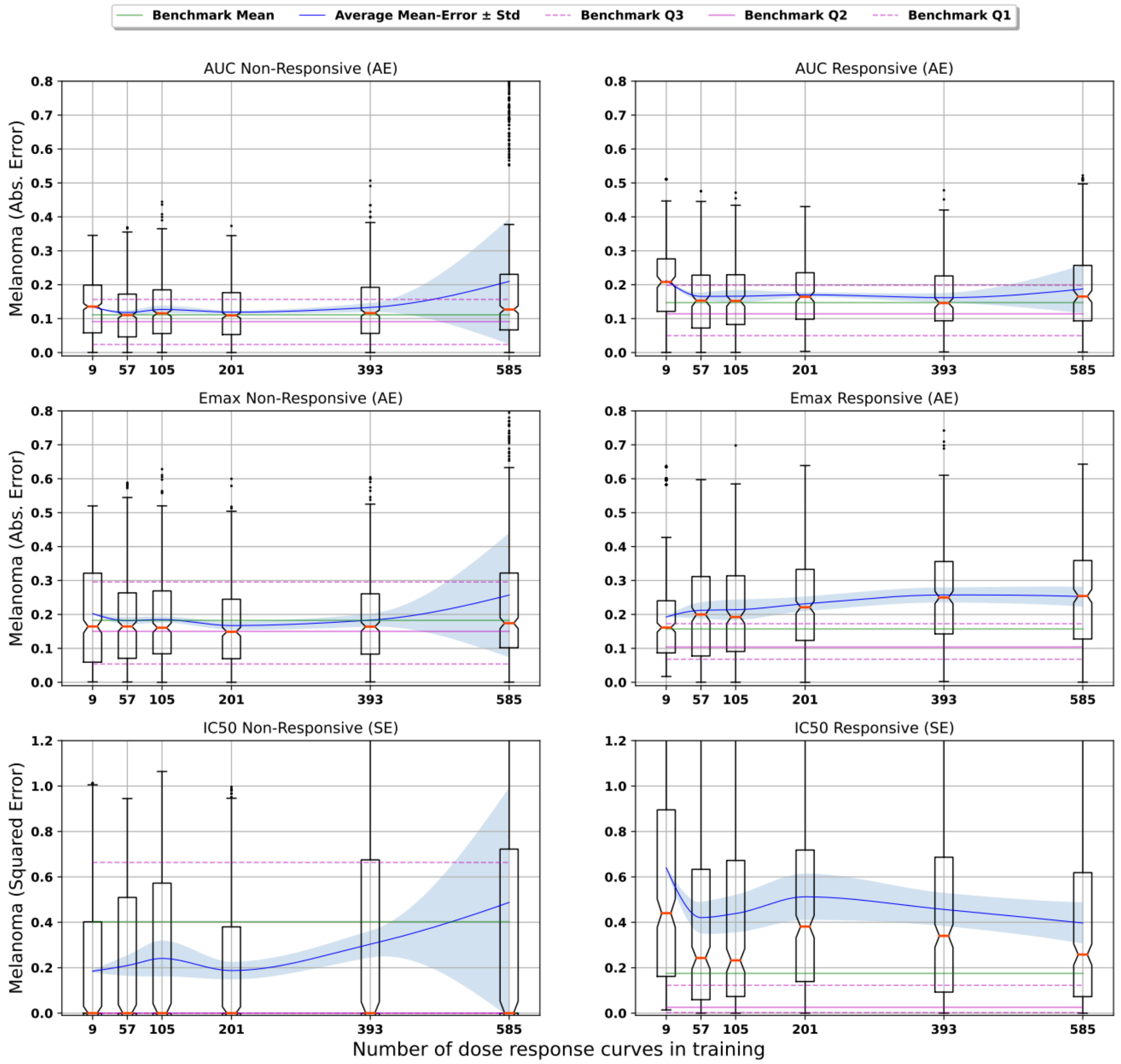

Supplementary Figure 15. Predictive performance of summary metrics over Melanoma cancer when increasing the dose response curves in training from the four remaining cancers: Breast, LUAD, COREAD and SCLC. Each subplot figure has a box-plot per value of increment showing the performance distribution of all MOGP models trained with six different seeds of random data. The blue line (with plus and minus a standard deviation) represents the average of the six mean-errors achieved by each of the models for the different random seeds. Additionally, the figures display a benchmark metric obtained from the error between the GDSC1 and GDSC2 datasets: the benchmark Mean (green solid line); the first quartile error Q1 (magenta dash line), second quartile error Q2 (magenta solid line) and third quartile error Q3 (magenta dash line).

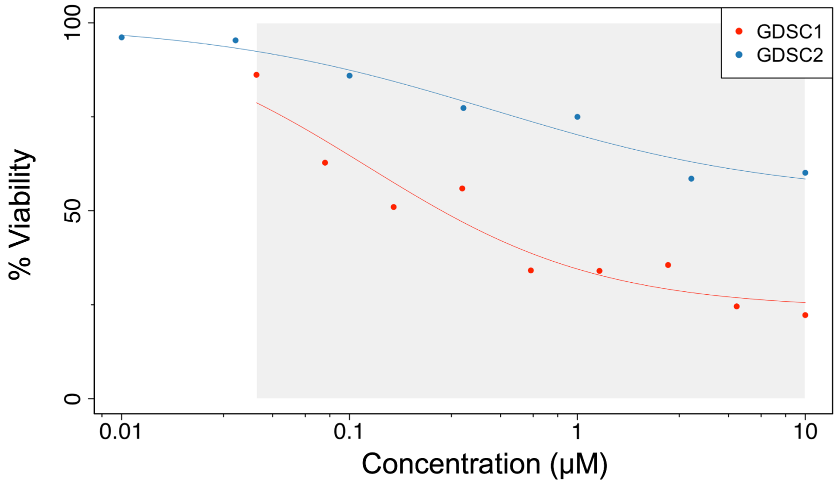

Supplementary Figure 16. This is an example of different drug concentrations used in GDSC1 ($D = 9$) and GDSC2 ($D = 7$) screenings for PLX-4720 treatment on A101D cells, with the shaded area indicating the overlapping dose-response region between the two datasets.

Supplementary References

1. Rahman, R., Dhruba, S. R., Ghosh, S. & Pal, R. Functional random forest with applications in dose-response predictions. *Sci. Rep.* **9**, 1628 (2019).
2. Wang, L., Li, X., Zhang, L. & Gao, Q. Improved anticancer drug response prediction in cell lines using matrix factorization with similarity regularization. *BMC Cancer* **17**, 513 (2017).
3. Ammad-Ud-Din, M. *et al.* Drug response prediction by inferring pathway-response associations with kernelized Bayesian matrix factorization. *Bioinformatics* **32**, i455–i463 (2016).
4. Ammad-ud-din, M. *et al.* Integrative and personalized QSAR analysis in cancer by kernelized Bayesian matrix factorization. *J. Chem. Inf. Model.* **54**, 2347–2359 (2014).
5. Liu, Q., Hu, Z., Jiang, R. & Zhou, M. DeepCDR: A Hybrid Graph Convolutional Network for Predicting Cancer Drug Response. *Bioinformatics* **36**, i911–i918 (2020).
6. Nguyen, T.-T., Nguyen, G. T. T., Nguyen, T. & Le, D.-H. Graph convolutional networks for drug response prediction. *IEEE/ACM Trans. Comput. Biol. Bioinform.* **19**, 146–154

(2022).

7. Cheng, X. *et al.* NeRD: a multichannel neural network to predict cellular response of drugs by integrating multidimensional data. *BMC Med.* **20**, 368 (2022).
8. Lundberg, S. M. & Lee, S.-I. A unified approach to interpreting model predictions. in *Proceedings of the 31st International Conference on Neural Information Processing Systems* 4768–4777 (Curran Associates Inc., Red Hook, NY, USA, 2017).
9. Byers, L. A. & Rudin, C. M. Small cell lung cancer: where do we go from here? *Cancer* **121**, 664–672 (2015).
10. Umemura, S. *et al.* Therapeutic priority of the PI3K/AKT/mTOR pathway in small cell lung cancers as revealed by a comprehensive genomic analysis. *J. Thorac. Oncol.* **9**, 1324–1331 (2014).
11. George, J. *et al.* Comprehensive genomic profiles of small cell lung cancer. *Nature* **524**, 47–53 (2015).
12. Álvarez, M. A. & Lawrence, N. D. Computationally efficient convolved multiple output Gaussian processes. https://www.jmlr.org/papers/volume12/alvarez11a/alvarez11a.pdf (2011).
